# Autoantibodies in cancer: a systematic review of their clinical role in the most prevalent cancers

**DOI:** 10.3389/fimmu.2024.1455602

**Published:** 2024-08-21

**Authors:** Ana Montero-Calle, María Garranzo-Asensio, Maria Teresa Moreno-Casbas, Susana Campuzano, Rodrigo Barderas

**Affiliations:** ^1^ Chronic Disease Programme (UFIEC), Instituto de Salud Carlos III, Madrid, Spain; ^2^ Investén-isciii, Instituto de Salud Carlos III, Madrid, Spain; ^3^ Biomedical Research Center Network for Frailty and Healthy Ageing (CIBERFES), Instituto de Salud Carlos III, Madrid, Spain; ^4^ Departamento de Química Analítica, Facultad de CC. Químicas, Universidad Complutense de Madrid, Madrid, Spain

**Keywords:** humoral immune response, autoantibodies, cancer autoantibodies, colorectal cancer, breast cancer, lung cancer, prostate cancer, proteomics

## Abstract

Although blood autoantibodies were initially associated with autoimmune diseases, multiple evidence have been accumulated showing their presence in many types of cancer. This has opened their use in clinics, since cancer autoantibodies might be useful for early detection, prognosis, and monitoring of cancer patients. In this review, we discuss the different techniques available for their discovery and validation. Additionally, we discuss here in detail those autoantibody panels verified in at least two different reports that should be more likely to be specific of each of the four most incident cancers. We also report the recent developed kits for breast and lung cancer detection mostly based on autoantibodies and the identification of novel therapeutic targets because of the screening of the cancer humoral immune response. Finally, we discuss unsolved issues that still need to be addressed for the implementation of cancer autoantibodies in clinical routine for cancer diagnosis, prognosis, and/or monitoring.

## 1 Introduction

The first reports describing the existence of a link between an immune response and cancer through autoantibodies were published in the 1950s. Since then, there has been growing interest in cancer autoantibodies. They are not only used as biomarkers to indicate the occurrence and development of cancer, disease response, and progression (1–3), but also to characterize the humoral immune response. This response is useful for identifying druggable targets and developing new cancer therapies. These therapies include potential immunotherapy drugs, tumor-infiltrating B cells, and stimulation of the intrinsic humoral immune system with radiation in metastatic disease, known as the abscopal effect (4–7).

**Figure 1:**
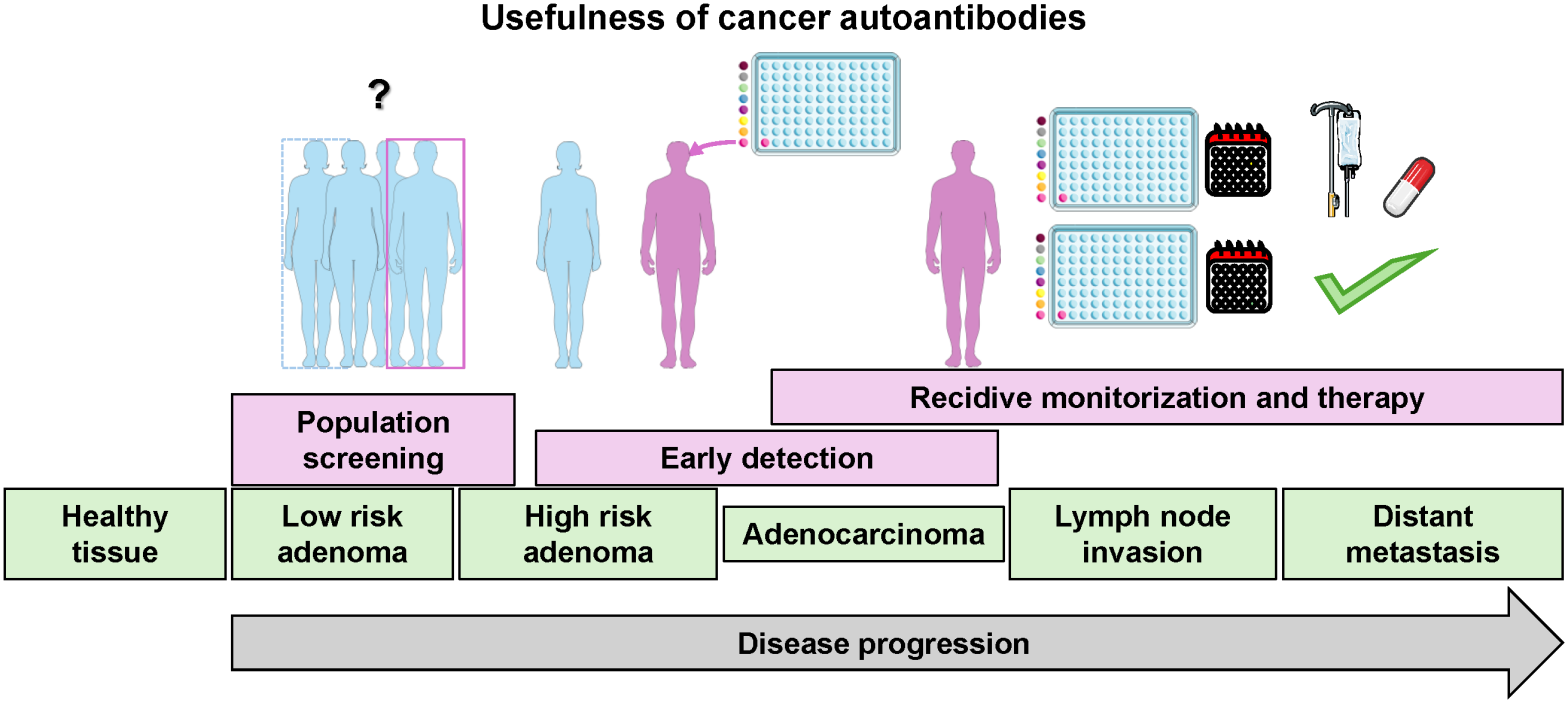
Schematic of the different uses of autoantibody detection according to cancer stages. The image was partially created using Servier Medical Art (https://smart.servier.com, accessed on February 26, 2024), provided by Servier, licensed under a Creative Commons Attribution 3.0 unported license.

This humoral immune response acts against self-proteins that are altered during the formation and progression of cancer. These alterations in self-proteins can include point mutations, frameshifts, chimeras, differential protein expression, or aberrant modifications, such as aberrant glycosylation, aberrant degradation, hyper-activation, and altered post-translational modifications, among other changes (8, 9). This autoimmune response to cancer occurs early in tumor formation and progression. As a result, the detection of blood autoantibodies should enable earlier cancer diagnosis than other techniques. Studies using murine models of colorectal cancer showed that a very early immune response against cancer lesions requires only a few tumor cells for local antigen processing by the immune system and the production of detectable cancer autoantibodies in serum samples useful for diagnosis (10). Although the role of autoantibodies in cancer is largely unclear, they have been suggested to have a cancer-promoting role, an antitumor effect, or to be an epiphenomenon associated with inflammation and tumor progression (11). In addition, autoantibodies are useful for cancer screening and preclinical diagnosis because their measurement in plasma or serum samples includes easy and minimally invasive sample collection, making their identification and validation a major approach in biomarker discovery in recent years (**Figure 1**).

Since the first reports identifying tumor-associated autoantigens (TAAs) as targets of cancer autoantibodies (TAAbs) in melanoma (12–14), technology has significantly evolved. Novel high-throughput approaches based on protein, phage, and peptide microarrays, phage display, next-generation sequencing (NGS), and mass spectrometry methods have been developed and applied for the discovery of novel autoantibodies and their respective TAAs in recent years. These advances in high-throughput strategies have increased the number and quality of candidate TAAs and facilitated the identification and validation of cancer-specific autoantibodies (15–23).

In this context, in this systematic review, we describe these novel methodologies for the identification and validation of cancer autoantibodies. We focus on those autoantibodies specific to the most common cancers worldwide (breast cancer -BC-, colorectal cancer -CRC-, lung cancer -LC-, and prostate cancer -PC-). These should be useful in obtaining validated autoantibody profiles for clinical use. We also discuss the potential of the protein targets of cancer autoantibodies as therapeutic intervention targets.

## 2 Methodologies for the identification and validation of cancer autoantibodies

The initial studies that identified cancer autoantibodies and their corresponding target autoantigens were published in the late 1970s and early 1980s. These studies utilized autologous serologic typing to identify circulating melanoma autoantibodies, using cultured tumor cells and sera from the same patients (12–14).

**Figure 2:**
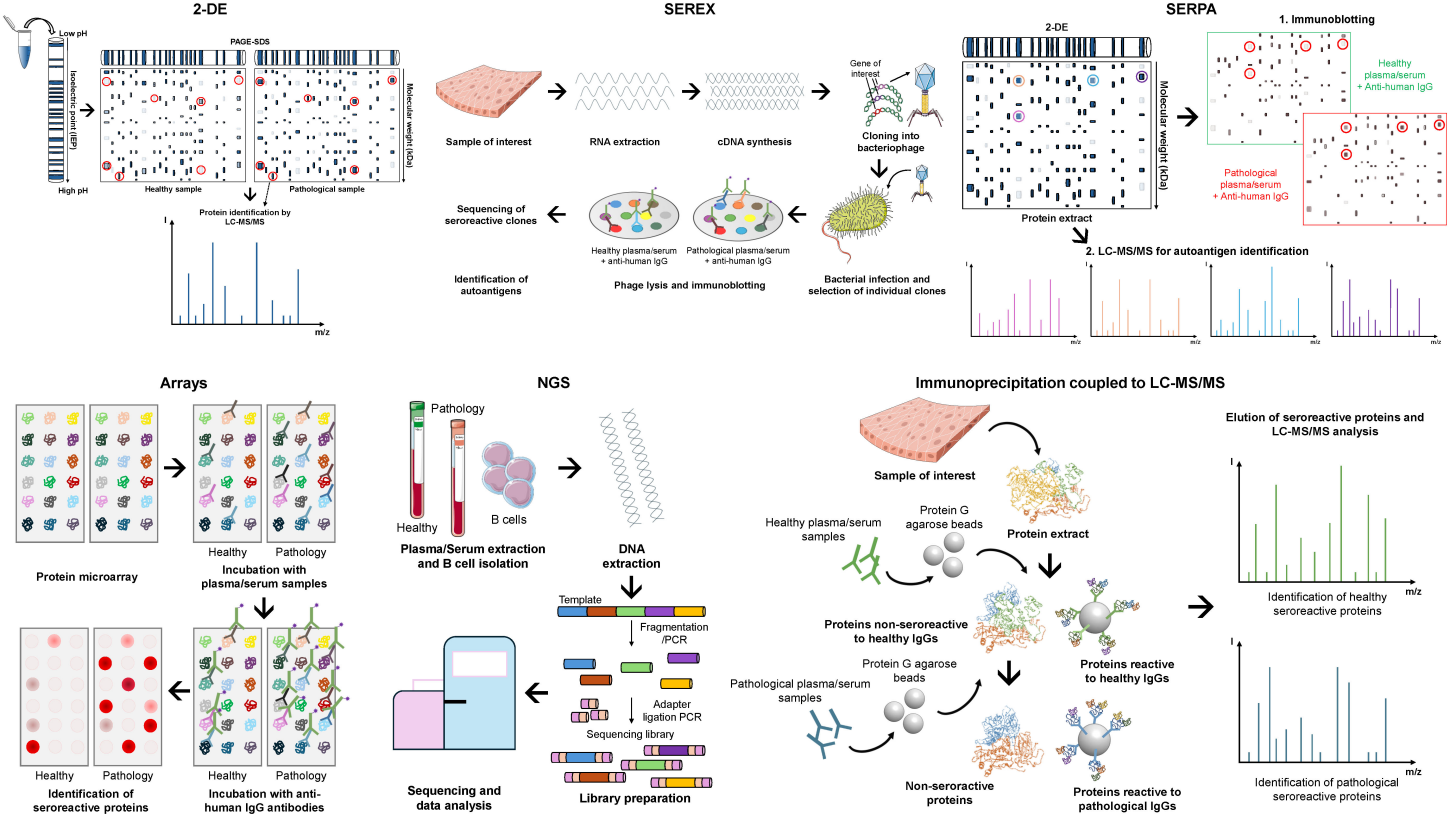
Screening methods for the identification of novel autoantigens and autoantibodies as blood-based cancer biomarkers. Representative workflow of the most widely used screening methods: *2-DE* (two-dimensional gel electrophoresis) coupled to liquid chromatography tandem mass spectrometry (LC-MS/MS) for protein identification; *SEREX* (serological analysis of tumor antigens by recombinant cDNA expression cloning), which combine cDNA library generation, cloning, and protein expression in bacteriophages (i.e. M13 phage) as fusion proteins to the bacteriophages’ capsid, and transference to a membrane for incubation with the samples of interest; *SERPA* (serological proteome analysis), which combine 2-DE, protein transference to nitrocellulose membrane for incubation with samples of interest, and a second 2-DE coupled to LC-MS/MS for the identification of seroreactive proteins; *Arrays*, consisting of commercial or custom-made protein microarrays containing the proteins of interest, which are incubated with the samples of interest; *NGS* (Next-generation sequencing), which consists of the isolation and fragmentation of B cells’ DNA for sequencing to identify seroreactive candidates specific of patients; and *Immunoprecipitation coupled to LC-MS/MS*, which consists of the isolation of patient’s seroreactive proteins and LC-MS/MS for protein identification.

Since then, several low-to-medium and high-throughput methods have been applied to different cancers. These include 2-DE (two-dimensional gel electrophoresis), SEREX (serological analysis of tumor antigens by recombinant cDNA expression cloning), SERPA (serological proteome analysis), NGS, and immunoprecipitation coupled to liquid chromatography tandem mass spectrometry (LC-MS/MS) (**Figure 2**). For 2-DE, proteins from the cancer of interest are separated by their isoelectric point (first dimension) and, subsequently, by their molecular weight (second dimension). Then, the most intense spots in pathological samples in comparison with healthy samples are selected as potential autoantigens, and the corresponding proteins identified by LC-MS/MS. Finally, the seroreactivity of candidate autoantigens must be validated by complementary techniques. For SEREX analyses, a cDNA library is generated from RNA extracted from the source of interest, which is then cloned in a bacteriophage phagemid as fusion proteins to the bacteriophages’ capsid (i.e. M13 phage). Then, recombinant phages are used to infect *Escherichia coli* bacteria cultures, which allow for the expression and the display of the recombinant proteins by the phages. Finally, these proteins are transferred to a membrane, and incubated with healthy and pathological plasma/serum samples to identify the seroreactive clones. Sequencing of positive clones is required to identify the seroreactive proteins. Finally, for SERPA analyses, after a first 2-DE with the protein extracts of interest, proteins are transferred to a nitrocellulose membrane and incubated with plasma/serum samples from healthy individuals and patients, followed by the incubation with a horseradish peroxidase (HRP) or fluorescence labeled anti-human IgG antibody (Ab). Then, a second 2-DE is performed with the same samples and the most seroreactive spots are isolated to identify the reactive proteins by LC-MS/MS. Despite the use of SEREX and SERPA over the years, these techniques have their challenges. The main issue with SEREX is the identification of mimotopes that have no sequence identity to known proteins but mimic the epitope of an altered protein. This makes the identification of the actual target protein of autoantibodies challenging. SERPA, on the other hand, tends to identify high- or medium-abundant proteins due to the sensitivity of the technique, but it allows for the identification of post-translational modifications (PTMs) and proteoforms autoantigens. However, SEREX and SERPA remain viable economic alternatives for research groups with limited budgets.

In recent years, the use of SEREX and SERPA has declined due to the superior performance of protein arrays, NGS, and mass spectrometry methods. Protein arrays consist of commercial or custom-made arrays containing the proteins of interest, which are then incubated with plasma/serum samples from healthy individuals and patients. Subsequently, the incubation of arrays with an HRP- or fluorescence-labeled anti-human IgG antibody reveals the most seroreactive spots to patients’ samples in comparison with healthy individuals, which are then selected as candidate cancer autoantigens. For NGS, B cells are isolated from plasma/serum samples from healthy individuals and patients. Then, the DNA is isolated and fragmented into manageable sizes. These fragments are then ligated to adapters to generate the sequencing library, and thus individual DNA molecules are sequenced. After a sequencing run, each sequence read is aligned to the reference genome to identify seroreactive candidates specific of patients. Finally, mass spectrometry methods for the identification of seroreactive autoantigens are mainly based on immunoprecipitation coupled to LC-MS/MS. Proteins from the source of interest are first incubated with IgGs from healthy individuals previously anchored to Protein G agarose beads to remove non-specific reactive proteins. Subsequently, non-reactive proteins (supernatants) are incubated with IgGs from patients previously anchored to Protein G agarose beads. Then, seroreactive proteins to both healthy and pathological IgGs are eluted and analyzed by LC-MS/MS to identify potential autoantigens specific of the disease.

Protein microarrays are a valuable tool for autoantibody discovery. Their key advantages include minimal sample and reagent consumption, and that the proteins under study are known in advance, which facilitates subsequent result verification (24). Currently, there are commercially available high-density protein microarrays (HUProt) containing about 21,000 human proteins, isoforms, and fragments from 16,794 unique genes. Of these, 15,889 are among the 19,613 canonical human proteins described in the Human Protein Atlas, making their use primarily limited by laboratory budget (22, 25–27). In addition to these protein microarrays, there are also microarrays of protein fragments that essentially cover the entire proteome, with one protein fragment of each gene (21, 23). This last alternative has been developed in recent years by the Human Protein Atlas platform (www.proteinatlas.org), which has produced antibodies and protein fragments -PrESTs (Protein-epitope signature tags)- for almost every human protein (without distinguishing between their isoforms) (28, 29). Phage microarrays provide an inexpensive, homemade alternative to commercial protein microarrays for discovering pathology-specific autoantibodies and their respective TAAs. This approach, which has been successfully used in various diseases, especially in cancer (30–32), involves enriching phage libraries, typically T7 phage, which display peptides of a specific pathology on their surface using sera/plasmas from pathological patients compared to controls. Individual phages are then printed on nitrocellulose slides and screened with patient and control sera or plasma to identify those phages that present the specific immunoreactive peptides (autoantigens) to a specific pathology. Regarding the limitations of protein microarrays, despite the potential to print the entire proteome on protein microarrays, considering one protein per gene without the presence of proteoforms of the same protein, the highest densities achieved so far are about 80% of the human proteome for recombinant protein microarrays and about 90% for PrESTs protein microarrays.

Regarding cons of protein microarrays, some authors have raised concerns that the 3D structure of the proteins might be lost during printing, which is especially important for PrEST protein microarrays where 3D non-linear relevant epitopes might be lost during screening since medium to large peptides without equivalent 3D structure to that of their corresponding native proteins are printed in this approach. In this context, another approach that combines immunoprecipitation directly coupled to LC-MS/MS has attempted to overcome these limitations. As autoantigens are immunoprecipitated in solution using IgGs isolated from patients and healthy individuals’ sera or plasma samples, and no SDS-PAGE gels are used for the identification of TAAs, the reactive proteins maintain their 3D conformation. This is crucial for the identification of autoantibodies against 3D discontinuous epitopes that might be missed in other approaches (19, 20, 22, 23, 33–36), potentially allowing for the completion of the cancer autoantibodyome.

## 3 Validation techniques for cancer autoantibodies

Usually, there is a poor coincidence between different studies. This discrepancy in the identification of TAAs may be attributed to several variables. These include the varying sensitivities of the techniques and platforms used for screening, the different protein repertoires printed on the microarrays, different expression systems for protein production, different PTMs in the proteins printed on microarrays relative to the cancer forms, or the use of different cancer cohorts from various populations (i.e., Asian, African, American, Hispanic, etc.), which could indicate the existence of cancer autoantibodies specific of ethnicity.

**Figure 3:**
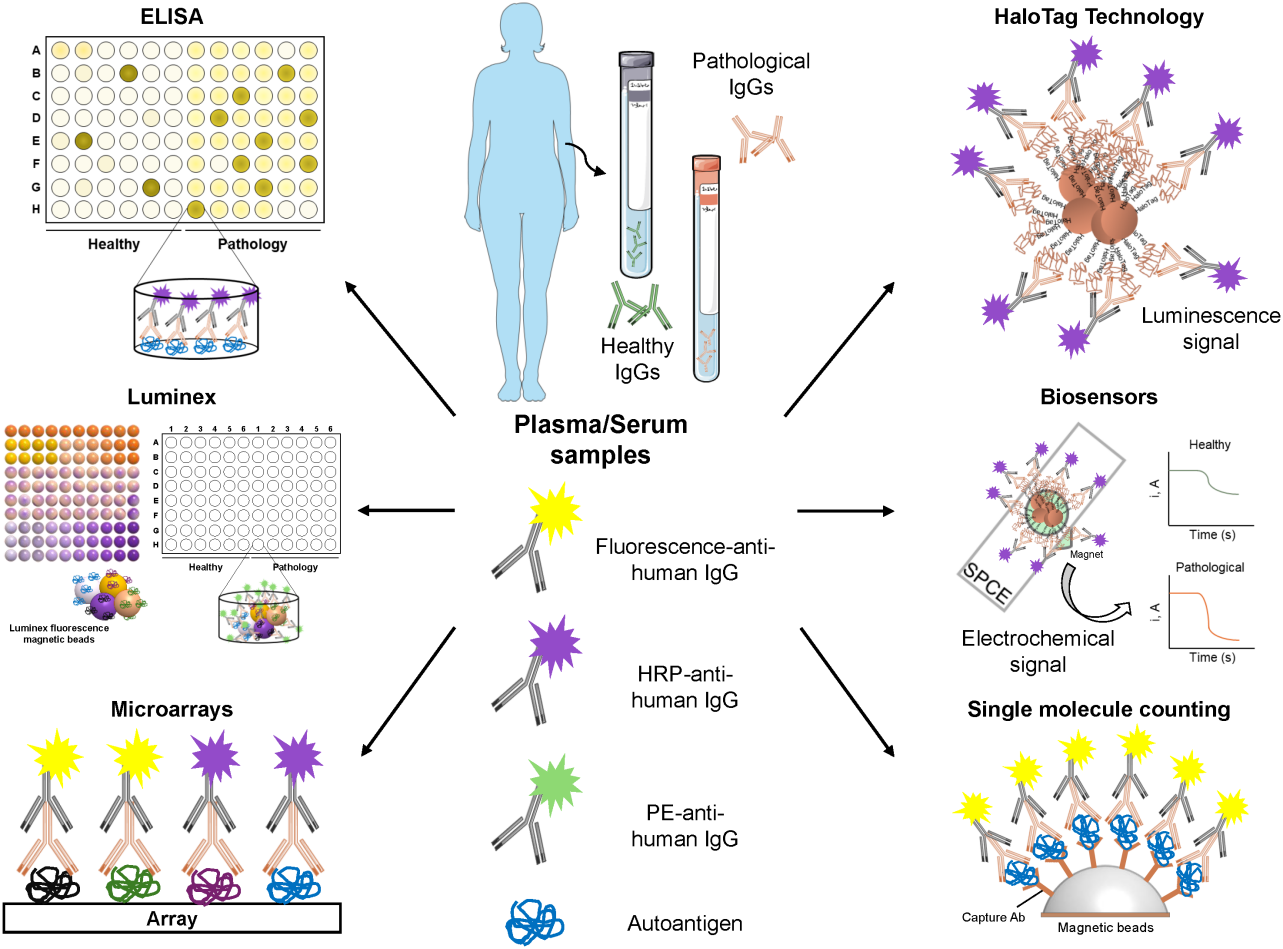
Validation methods for the identification of novel autoantigens and autoantibodies as cancer blood-based biomarkers. Representative workflow of the most widely used methods for the validation of the seroreactivity of candidate autoantigens. Once autoantigens have been selected, their seroreactivity must be confirmed in a larger cohort of plasma/serum samples from healthy individuals and patients using different techniques, such as: ELISA (enzyme-linked immunosorbent assay), by coating the plates with candidate autoantigens *in vitro* expressed or expressed in mammalian, bacterial, or insect cells; Microarrays with candidate autoantigens printed on their surface; Luminex, consisting in the immobilization of candidate autoantigens fused to a tag (i.e. 6xHis or HaloTag) onto a specific color-coded magnetic bead previously activated with the corresponding ligand; HaloTag technology, in which candidate autoantigens are cloned as HaloTag fusion proteins for their covalent anchored to magnetic beads coated with a Haloalkane (HaloTag ligand); Biosensors, which consist of the capture of HaloTag fusion proteins immobilized on magnetic beads on the surface of screen-printed carbon electrodes (SPCEs) and allows the development of electrochemical signals in presence of the hydroquinone (HQ)/H_2_O_2_ system; and Single molecule counting (SIMOA/SMCx), ultrasensitive ELISAs that used capture Ab-coated beads for the anchoring of the autoantigens of interest. Quantification is possible if a standard curve is performed. Some of the image elements were created using Servier Medical Art (https://smart.servier.com), accessed on February 26, 2024, provided by Servier, licensed under a Creative Commons Attribution 3.0 unported license.

Many of these issues could be addressed by performing validation assays with independent patient’s cohorts that differ from those used in the discovery phase, and by including samples from various ethnic groups. Although this validation is still lacking in many reports, several approaches have been developed for the validation of cancer autoantibodies and their protein targets (**Figure 3**). These include ELISA (enzyme-linked immunosorbent assay), low-density protein microarrays, Luminex, HaloTag-based immunoassays, HaloTag-based biosensors, and single molecule counting approaches, such as SIMOA or SMCx (19, 20, 36–40).

Validation experiments should be conducted with the highest quality proteins, which mimic the actual target as closely as possible. In this regard, HaloTag fusion proteins represent a good alternative to other methodologies. HaloTag allows for their covalent binding to any Haloalkane-modified surface (20, 38, 41), and fusion proteins can either be expressed *in vitro* or *in vivo* in a mammalian environment. This means that the highest quality tumor-associated antigen becomes oriented in the assay surface, allowing for a better recognition of the autoantibodies to their target proteins. This contrasts with ELISA or Luminex, where the proteins can be partially denatured as a result of immobilization to the plates or to the covalent immobilization to the Luminex magnetic beads (37, 39). However, Luminex allows for the simultaneous detection and quantification of multiple autoantigens (up to 500) at the same time and in a single experiment, thus reducing the amount of sample required and the time of analysis, which makes this technique an interesting tool for the detection of autoantibodies. Additionally, SIMOA or SMCx single molecule counting approaches are less explored alternatives that should be considered for the validation of cancer-specific autoantibodies. Meanwhile, biosensors represent a really interesting alternative as point-of-care (POC) and affordable devices for their implementation into clinical routine (20, 38, 41, 42).

Finally, ROC (Receiver Operating Characteristic) curves are useful for the determination of the diagnostic ability of candidate biomarkers or a panel of biomarkers, as they provide information about the probability to classify a patient as a diseased subject and a healthy individual as a non-diseased subject (area under the curve -AUC-), and the ability of the test to detect true positives (sensitivity), and true negatives (specificity). Thus, those autoantibodies showing higher AUCs, sensitivities, and specificities are the best diagnostic biomarkers of a disease (43, 44). In addition, diagnostic panels including several autoantibodies are needed to increase the capacity of the test to discriminate between healthy individuals and patients, as not all patients might possess autoantibodies against all autoantigens. Thus, multiplexed bioplatforms for the simultaneous detection of these autoantibodies in plasma or serum are of high interest.

## 4 Humoral immune response for the detection of the four most incident cancers worldwide

Autoantibodies in serum or plasma of healthy and pathological individuals are mainly IgM and IgG isotypes. Natural IgM autoantibodies are produced by a mechanism of positive selection of self-antigens to clean-up cellular debris, and to maintain the tissue and immune homeostasis. Most self-reactive IgMs are polyreactive and possess a moderate affinity to their antigens (45, 46). On the contrary, IgGs autoantibodies are high affinity antibodies produced as a consequence of a breakdown in self-tolerance due to a pathology (46–48). Because self-reactive IgGs reflect a pathological process, the use of autoantibodies for cancer diagnosis or prognosis is focused on the identification of IgGs in human samples.

In this context, the most widely known cancer autoantibody is that produced against p53. Autoantibodies against p53 have been widely described in different types of cancer, such as CRC, BC, ovarian, or gastric cancers, among others, and their production has been mainly associated to missense mutations and p53 accumulation in cancer patients (49, 50). Despite the high specificity of p53 autoantibodies (> 96%), they possess a very low sensitivity (< 35%), as not all cancer patients show high serum or plasma levels of autoantibodies against this protein. Thus, autoantibody panels and their target proteins have been identified and investigated as diagnostic or prognostic tools for different cancer types.

In this review, validated autoantibodies with promising diagnostic potential, those appearing in at least two different reports with high diagnostic ability, or those autoantibodies with prognostic potential of the four most common cancers worldwide (breast, lung, colorectal, and prostate) have been summarized.

### 4.1 Autoantibodies in breast cancer

Breast cancer (BC) is the most common cancer worldwide, with a 12.5% global incidence in both genders in 2020, and the fifth cause of cancer-related death worldwide (51). According to the World Health Organization (WHO), male BC accounts only for < 1% of total cases. In the last years, deaths associated to BC have significantly decreased due mainly to early diagnosis by mammography imaging. However, the sensitivity of this technique is reduced to 50% in women with dense breasts, which is a main characteristic of most BCs. Despite the high sensitivity of magnetic resonance imaging (MRI), even in dense breast tissues (90-93% sensitivity), this technique is not recommended for screening due to its low specificity, resulting in numerous false positive cases. Thus, MRI for early screening is recommended only in women at a high risk of BC (e.g. reproductive and hormonal risk factors, lifestyle risk factor, or genetic predisposition) (52–54). The treatment of BC patients depends on the molecular subtype, the tumoral burden, and the risk of recurrence. Breast-conserving surgery is always the first option, but mastectomy can be oncologically required even for prevention. In addition, neoadjuvant or adjuvant therapies might be also recommended, based on chemotherapy or endocrine therapies, and radiotherapy before or after surgery or mastectomy is sometimes needed to reduce the risk of recurrence and BC mortality (52, 53).

Since the first report in 1982 on BC autoantibodies, different autoantigens have been identified by different techniques, including ELISA, phage or protein microarrays, or nucleic acid programmable protein arrays (NAPPA) (55–58). Additionally, it has been described that the presence of these autoantibodies in the blood of BC patients can be detected long before their target autoantigen. Among the 26 individual autoantigens of BC described to date (55–58), only 11 (MUC1 (CA 15.3), IMP2/p62, HSP60, Her2/Neu, Survivin, CDKN2A (p16), c-MYC, BRCA1, BRCA2, Cyclin B1, and NY-ESO-1 -which have been also described to be overexpressed in BC tissue samples-) out of them have been described as BC autoantigens in two or more studies, although their diagnostic ability (AUC, sensitivity, and specificity) was not calculated in all of them (**Table 1**) (58–61, 64–75).

**Table 1:** Individual autoantibodies described in BC with diagnostic ability of the disease.

Autoantigen	AUC (%)	Sensitivity (%)	Specificity (%)	Reference/s
p53	63-68	34.9	90.0	(59–63)
MUC1 (CA 15.3)	72-78	11-20	96-98	(64, 65)
IMP2/p62	65.1	–	–	(59, 66)
HSP60	63.7	31.8	95.7	(61, 67, 68)
Her2/Neu	60.0	17-18	94.0	(60, 69, 70)
Survivin	59.0	21.8	90.0	(59, 71–73)
CDKN2A (p16)	74.0	30.3	90.0	(74, 75)
c-MYC	–	13-22	97-100	(59, 60)
BRCA1	–	8.0	91.0	(57, 58)
BRCA2	–	34.0	92.0	(57, 58)
Cyclin B1	68.0	–	–	(60, 61)
NY-ESO-1	–	26.0	94.0	(57, 60)
HNRNPF	72.0	84.2	60.8	(76)
FTH1	68.0	81.2	56.1	(76)
TOPO48	80.1	100	76.0	(77)

Serum levels of autoantibodies against TOPO48, described by He et al. in 2020 by ELISA, possess the best individual diagnostic ability to discriminate early BC patients from healthy and benign breast disease (BBD) individuals, with an AUC, sensitivity, and specificity of 80.1%, 100%, and 76%, respectively (77). In addition, autoantibodies against HNRNPF and FTH1 were also identified in BC by phage display and ELISA, showing these autoantibodies, together with autoantibodies against MUC1, one of the best individual BC diagnostic abilities -higher than 65%- with sensitivities and specificities higher than 80% and 55%, respectively (76). However, autoantibodies against TOPO48, HNRNPF, and FTH1 in BC have been only described in one study, and thus, more research is needed to establish this biomarker panel for BC clinical diagnosis (**Table 1**). In contrast, the other individual autoantibodies described previously possess very low diagnostic abilities and sensitivities (lower than 60% and 34%, respectively), but high specificities (higher than 90%).

Among these autoantibodies, MUC1, Her2/Neu, c-MYC, and NY-ESO-1 autoantibodies have been demonstrated to be useful as early diagnostic biomarkers of BC. Nevertheless, autoantibodies against MUC1 and c-MYC were described to significantly discriminate not only BC patients from healthy individuals but from patients with BBD. Additionally, autoantibodies against p53 have also been described in BC, but with a lower sensitivity than that of other autoantibodies previously described (62, 63). However, higher levels of p53 autoantibodies have been mainly associated with a worst prognosis of the disease than with an early stage of BC and to many different cancers, as well as NY-ESO-1 autoantibodies, and thus, the individual measurement of these autoantibodies in plasma or serum is not enough for the specific diagnosis of BC.

Due to the low diagnostic ability of identified individual autoantibodies, most studies have been focused on the study of panels of autoantibodies for the diagnosis of BC (**Table 2**). It has been demonstrated that the combination of most of them significantly increase the diagnostic ability of the disease. In the last years, more than 25 panels of BC autoantibodies have been described. The combination of the previously described individual autoantigens significantly increased their sensitivity for the diagnosis of the disease to 43.9% (CDKN2A, c-MYC, p53), 64% (p53, c-MYC, NY-ESO-1, BRCA2, Her2/Neu, MUC1), or 89.3% (FTH1, HNRNPF, MUC1) (58, 74, 76). However, other authors have performed different strategies, such as NAPPA arrays or 2-DE gel analysis coupled to mass spectrometry, and ELISA, for the identification of diagnostic panels of autoantibodies. As a result, large autoantibody panels (combining 5, 13, or 28-autoantibodies) with promising characteristics for early BC diagnosis have been described (78–80). In addition, in 2017, eleven of these autoantibodies were selected for clinical trials to develop a test for the daily diagnosis of BC in clinical routine by liquid biopsy (82). As a consequence of the high sensitivity and specificity obtained during the trials (87.5% sensitivity and 83.8% specificity), the Videssa Breast test, combining the detection of 11 proteins and 33 autoantigens, was launched in 2023 in the United States as a tool for the blood detection of BC, irrespective of breast density, with a 98% accuracy (81, 83).

**Table 2:** Autoantibody panels reported in more than one study for the detection of BC patients with high sensitivity and specificity.

Autoantigen panel	AUC (%)	Sensitivity (%)	Specificity (%)	Reference/s
CDKN2A+c-MYC+p53	–	43.9	97.6	(74)
p53+c-MYC+NY-ESO-1 +BRCA2+Her2/Neu+MUC1 (early detection)	–	64	85	(58)
FTH1+HNRNPF+MUC1	93.1	89.3	93.8	(76)
GAL3+PAK2+PHB2+RACK1+RUVBL1	81	66	84	(78)
CTAG1B+CTAG2+TP53+RNF216+PPHLN1+PIP4K2C+ZBTB16+TAS2R8+WBP2NL+DOK2+PSRC1+MN1+TRIM21	68	33	98	(79)
ATP6AP1+PDCD6IP+DBT+CSNK1E+FRS3+RAC3+HOXD1+SF3A1+CTBP1+C15ORF48+MYOZ2+EIF3E+BAT4+ATF3+BMX+RAB5A+UBAP1+SOX2+GPR157+BDNF+ZMYM6 +SLC33A1+TRIM32+ALG10+TFCP2 +SERPINH1+SELL+ZNF510	75.6	80.8	61.6	(80)
Videssa Breast test (11 proteins + 33 autoantigens)	–	88-92	81-87	(81–83)

Regarding prognosis, autoantibodies against HER2, different MUC1 glycoforms, and SELENOP have been associated with BC patients’ prognosis. In this sense, high HER2 autoantibody levels have been described to possess a protective effect in BC, associated to a reduced risk of HER2^+^ BC subtype, and a better recurrence-survival rate after a 6-3686 day interval follow-up study of BC patients (84). Furthermore, high plasma autoantibody levels against two MUC1 glycoforms (core3MUC1 (GlcNAcβ1-3GalNAc-MUC1) and STnMUC1 (NeuAcα2,6GalNAc-MUC1)) have been described to reduce the incidence and increase the metastatic time in BC patients after a 15-year follow-up (64). In contrast, a 4-9 year follow-up study of BC patients associated high plasma levels of SELENOP autoantibodies with a high risk of poor prognosis of BC, with higher recurrence, and mortality rates (85). Moreover, high TOPO48 and p53 autoantibodies have been associated with a favorable prognosis at early stages and with shorted 5-year interval rates, respectively (77, 86). Finally, thyroid autoimmunity autoantibodies have been postulated as predictive parameters of BC. Specifically, higher levels of anti-TG and anti-TPO autoantibodies in BC patients have been related to a low risk of axillary involvement and of ki67 proliferation index of breast tumoral cells (87–89).

### 4.2 Autoantibodies in lung cancer

Lung cancer (LC) is the leading cause of cancer-related death worldwide mainly due to its late diagnosis and consequent poor prognosis (90–92). As with many cancer types, its 5-year survival rate greatly depends on the stage the cancer is diagnosed, evidencing the need to find new efficient early diagnostic tools. Particularly, when diagnosed early (stage I), LC patients have reported 5-year survival rates between 75-60%, while advanced LC patients’ 5-year survival rate reports are of approximately 15-5% (93, 94). However, most LC patients (57% of all cases) are diagnosed when they have developed metastasis while only 15% of all cases correspond to patients with localized disease (94). Currently, low-dose computed tomography (LDCT) is used as a LC diagnostic tool. This screening approach shows great sensitivity for eligible patients, but it also produces a high number of false-positive cases (95). Thus, the particularities of autoantibodies make them an interesting diagnostic tool choice, and several studies have been focused on finding a diagnostic signature that could aid in the management of the disease.

A previous study found 67 reported works that evaluated the diagnostic capability of either single or multiple autoantibodies for LC (96). On the other hand, another study focusing on relevant data from more than three reports, found 53 relevant articles through which they evaluated the use of single autoantibodies against p53, c-MYC, Survivin, NY-ESO, Cyclin B1, CAE, GBU 4-5, p16, HuD, and SOX2 (97) (**Table 3**). Unsurprisingly, the use of single autoantibodies showed low sensitivity for LC detection, while the use of multiple autoantibodies could detect LC with high sensitivity and specificity differently according to LC stage, making the stratification of patients possible (98, 99) (**Table 4**). Among the single autoantibodies described for LC, NY-ESO-1 has shown promising results since, despite its low sensitivity, it has been proven to have great specificity and its inclusion in different autoantibody panels or diagnostic signatures greatly increases their strength (99, 105, 106).

**Table 3:** Diagnostic performance of individual autoantibodies for LC detection (97).

Autoantigens	AUC (%)	Sensitivity (%)	Specificity (%)
LC vs controls			
p53	82	19	98
NY-ESO-1	90	17	98
Survivin	96	19	99
c-MYC	45	14	98
Cyclin B1	91	18	98
GBU4-5	91	7	98
CAGE	90	14	98
p16	91	8	97
SOX2	93	14	99
HuD	82	17	99

**Table 4:** Diagnostic performance of the combination of autoantibodies for LC detection.

Autoantigens	AUC (%)	Sensitivity (%)	Specificity (%)	Reference
LC vs controls				
p53, GAGE7, PGP9.5, CAGE, MAGEA1, SOX2, and GBU4-5	80.6	61.5	88.5	(92)
p53, GAGE7, PGP9.5, CAGE, MAGEA1, SOX2, and GBU4-5	–	61.5	88.5	(100)
p53, NY-ESO-1, CAGE, GBU4-5, Annexin 1, and SOX2	63	39	89	(101)
p53, NY-ESO-1, CAGE, GBU4-5, Annexin 1, and SOX2	64	37	90	(101)
p53, NY-ESO-1, CAGE, GBU4-5, Annexin I, and SOX2	–	39	89	(102)
p53, NY-ESO-1, CAGE, GBU4-5, SOX2, HuD, and MAGE A4	–	41	91-93	(102)
p53, NY-ESO-1, CAGE, GBU4-5, Annexin I, and SOX2	–	46	83	(103)
p53, NY-ESO-1, CAGE, GBU4-5, SOX2, HuD, and MAGE A4	–	37	91	(103)
p53, NY-ESO-1, CAGE, GBU4-5, Annexin 1, and SOX2	–	38	88	(104)

It is interesting to note that currently, the China Food and Drug Administration has approved a 7 autoantibodies (p53, GAGE7, PGP9.5, CAGE, MAGEA1, SOX2, and GBU4-5) kit for the specific detection of LC in the Chinese population using serum samples (100). Moreover, a recent study has evaluated the use of this particular kit alongside LDCT to diagnose cases worldwide (92). These researchers found that the TAAbs panel worked better than individual autoantibodies and had great specificity (88.5%) and promising sensitivity (61.5%). Furthermore, the combination of LDCT and TAAbs screening was a far better diagnostic tool than LDCT alone. When testing the 7 TAAbs panel in the real-world cohort, the panel showed comparable results for patients at stages I and II-IV regardless of gender or age, highlighting its usefulness as an early detection platform (92). Other studies have defined different signatures comprised of only autoantibodies or combination of autoantibodies and proteins. Remarkably, there are TAAbs common to some of the described signatures and the 7 autoantibody signature approved in China (p53, CAGE, GBU4-5) (101–103, 107), while other autoantibody signatures remain unique (107). Among the other diagnostic panels described, the EarlyCDT-Lung panel of 6 (p53, NY-ESO-1,CAGE, GBU4-5, Annexin I, and SOX2) or 7 (p53, NY-ESO-1, CAGE, GBU4-5, SOX2, HuD, and MAGE A4) autoantibodies released by Oncimmune Inc. showed high specificity and could detect elevated autoantibodies in 40% of LC (both non-small cell and small cell LC) independently of cancer stage (101–104, 108). Although the percentage of patients detected still needs improvement, the fact that it can detect LC regardless of its type and stage with the same strength makes the panel a promising tool for the screening of the disease. Regarding the diagnostic signatures based on the detection of proteins and autoantibodies, the PAULA (Protein Assay Using Lung Cancer Analytes) test from 20/20 Genesystems uses a panel of 4 TAA (CEA, CA-125, and CYFRA 21–1) and one autoantibody (NY-ESO-1) to discriminate between LC patients and controls with high sensitivity and specificity (109). Further studies validated the use of this panel for the discrimination of non-small cell LC from normal patients and patients with benign pulmonary diseases (99).

Despite the fact that most studies focused on the detection of the LC autoantibodies in serum or plasma samples, other studies have focused on their detection on other advantageous biological samples such as sputum, which is easy to collect and it is secreted directly from the lower airways and deep lungs where tumors reside, making it a direct source of autoantibodies (110). In sputum, autoantibodies against DDX6, ENO1, and 14-3-3θ could detect LC with high sensitivity (81%) and specificity (83%) independently of race, gender, and tumor stage and type; however, their combined detection with serum autoantibodies, other molecular markers, or LDCT was not reported, and further testing should be performed to evaluate the usefulness of this panel in a clinical setting.

Taken together, these results seem promising in the development of a non-invasive diagnostic tool based on the detection of autoantibodies capable of assisting the current methodologies in the early screening and recognition of LC patients. However, despite that there are at least three commercially available kits that could aid in the early detection of LC patients, their use in a medical setting is still limited and efforts should be made to encourage it.

Finally, regarding the prognostic ability of autoantibodies for LC, and although they have not been already established in clinical routine, there have been several studies focusing on their importance (111). In this sense, autoantibodies against p53, PGP9.5, SOX2, and MAGEA1 have been described to correlate with the overall survival of NSCLC patients (112), while a highly predicted autoantibody panel composed of 13 autoantibody biomarkers against SPATA19, TSPY3, GLS2, TCEA2, TSGA10, HMGN5, LUZP4, HDAC4, SPACA3, IMPDH1, TXN2, TFG, and PPP2R1A has been described to predict postoperative survival of LC patients (113).

### 4.3 Autoantibodies in colorectal cancer

Colorectal cancer (CRC) is the third most common cancer and the second cause of cancer-related death worldwide, whose development can take between 10 to 15 years. CRC development has been described as the adenoma-adenocarcinoma transition, as most CRC cases develops from polyps (premalignant lesions -low- and high-grade colorectal adenomas) restricted to mucosa that start to proliferate and invade different layers of the intestinal epithelium (adenocarcinoma) (114, 115). In addition, four different CRC stages have been described according to the size of the primary carcinoma and the spread of cancerous cells to other organs. Thus, CRC stages can be divided into early non-metastatic stages I and II, and advanced metastatic stages to lymphatic nodes (stage III) or to distal organs (stage IV), being the liver and lung the main organs of colonization of CRC metastasis. Because CRC is usually asymptomatic at early stages of the disease, currently most CRC patients (> 60%) are diagnosed at advanced stages of the disease, when the 5-year survival rate of patients decrease to 70-10% in comparison with its diagnosis at early stages (80-90%). In addition, the clinical symptoms of CRC (occult fecal blood and changes in the bowel habits) might be related to other pathologies not associated to cancer, thus delaying its diagnosis (116).

The current diagnosis of CRC involves an initial screening of fecal occult blood, which is not specific of the disease and is mainly associated to advances stages of CRC, and colonoscopy, which is a very sensitive but an invasive technique that requires bowel preparation and sedation, and thus, it cannot be performed routinely for diagnosis. Finally, surgery is always the first option for the treatment of the disease, which allows for the removal of all malignancies, whereas metastases are only resected in those patients with a good response to chemotherapeutic agents. In addition, chemotherapy and/or radiotherapy treatments might be also applied to patients after surgery to prevent for recurrences, and in cases of unresectable tumors to suppress the progression and growth of cancerous cells (117–119). Therefore, with the objective to improve the early diagnosis of CRC, novel biomarkers, and mainly blood based biomarkers that might be easily measured in clinical routine are mandatory.

Among the most common biomarkers of CRC (carcinoembryonic antigen (CEA), CA-19.9, and CA125), CEA protein has been widely described as a significant diagnostic and prognostic CRC biomarker (120–122). CEA expression is significantly higher in CRC tumoral tissues than in healthy tissues (< 60 times higher). In addition, these higher levels of CEA in tissues have been associated with a poor prognosis of the disease. Furthermore, CEA serum levels have been also found increased in CRC patients in comparison to healthy individuals, which was also associated with a poor prognosis of the disease, demonstrating that the measurement of CEA levels in serum is not useful for the early diagnosis of the disease, but as a prognostic biomarker of CRC (123–126). Due to the increased expression of CEA in CRC, autoantibodies against this protein have been also investigated in several works (127–129). Most of these studies were focused on CRC patients at advanced stages of the disease and healthy individuals, highlighting its high specificity (from 59.5% to 98%) but low sensitivity (from 21% to 63.8%) for advanced CRC diagnosis (**Table 5**).

**Table 5:** Individual autoantibodies described in CRC and individuals with premalignant lesions with diagnostic potential of the disease.

Autoantigen	AUC (%)	Sensitivity (%)	Specificity (%)	Reference/s
Diagnosis of CRC patients				
CEA	80-92	13-85	60-100	(127–129)
p53	50-69	15-62	13-100	(19, 20, 38, 126, 130)
p73	65.3	53.8	88.3	(126)
ΔNp73α	67.4	48.7	91.7	(126)
ΔNp73β	61.9	46.2	95.0	(126)
p53γ	61.3	64.5	58.3	(130)
Δ40p53β	67.5	93.5	41.7	(130)
Δ133p53γ	80.1	77.4	75.0	(130)
TAp63α	74.1	51.6	87.5	(130)
TAp63δ	73.7	58.1	79.2	(130)
ΔNp63α	74.7	80.6	64.6	(130)
ΔNp63δ	67.7	41.9	89.6	(130)
MUC1	–	7-48	83-95	(131, 132)
c-MYC	–	4-22	95-100	(74, 133–135)
Survivin	–	4-57	64-100	(135–137)
ANXA4	–	6-15	90-98	(138, 139)
p62	–	19-23	97-99	(134, 135, 140, 141)
RPH3AL	–	7-72	84-98	(138, 139, 142)
NY-CO-16	–	18-41	90-100	(138, 143, 144)
koc	–	9-15	98-100	(134, 135, 141)
HDAC5	–	3-22	90-100	(138, 144, 145)
IMP1	–	13-22	98-100	(134, 135)
NY-ESO-1	–	7-17	98-100	(145, 146)
MAGEA3	–	3-8	98-100	(139, 145)
Cyclin B1	–	16-33	98.0	(133, 135)
SEC61β	–	30-79	75-80	(138, 147)
CCCAP	–	22-35	90-96	(138, 144)
IMPDH2	–	8-32	98-100	(139, 148)
GRP78	–	16-20	100	(149, 150)
PIM1	64-85	13-64	83-93	(37–39, 128)
MAPKAPK3	73-77	31-73	74-90	(37–39, 128)
STK4	69-74	22-72	42-90	(30, 37–39, 128)
SRC	68-71	72-67	62-73	(38, 39, 128)
ACVR2B	66-67	59-60	76	(39, 128)
SULF1	63-69	50-88	47-79	(30, 38, 39)
TALDO1	65.4	48.4	90.0	(19, 20)
GTF2B	63-72	13-56	60-90	(37, 38)
ACTR3	66.9	68.4	65.8	(19, 20)
MT-CO2	67.7	63.2	65.8	(19, 20)
Phage-expressed peptide NHSL1	52-60	52-57	50-52	(30, 39)
Phage-expressed peptide SREBF2	53-61	48-55	61-70	(30, 39)
Phage-expressed peptide GRN	52-62	58-55	57-59	(30, 39)
Phage-expressed peptide GTF2i	57-60	58-60	52.0	(30, 39)
Diagnosis of individuals with premalignant lesions				
p53	53-61	36-53	80-92	(19, 20, 38, 126, 130)
p73	78.9	70.0	88.3	(130)
ΔNp73α	79.1	70.0	93.3	(126)
ΔNp73β	69.0	56.8	96.7	(126)
p53γ	69.5	54.8	83.3	(130)
Δ40p53β	69.5	64.5	70.8	(130)
Δ40p53γ	68.1	67.7	72.9	(130)
Δ133p53γ	81.7	90.3	77.1	(130)
Δ160p53γ	69.2	45.2	85.4	(130)
TAp63α	78.7	74.2	75.0	(130)
TAp63δ	79.1	71.0	79.2	(130)
ΔNp63α	81.0	74.2	79.2	(130)
ΔNp63δ	76.9	90.3	52.1	(130)
PIM1	85.5	76.9	93.3	(38)
MAPKAPK3	76.0	65.4	90.0	(38)
STK4	74.3	73.1	73.3	(38)
SRC	68.8	73.1	73.3	(38)
SULF1	70.4	88.5	46.7	(38)
TALDO1	71.9	83.3	63.3	(19)
GTF2B	70.3	53.8	83.3	(38)
ACTR3	66.6	69.4	65.8	(20)
RAB2A	66.9	38.9	92.1	(20)
MT-CO2	64.6	61.1	68.4	(20)

Since 1995, more than 80 studies regarding autoantibodies in CRC with diagnostic ability of the disease have been published, describing more than 200 proteins as potential autoantigens of the disease identified and validated by different techniques, such as SERPA, protein microarrays, or phage display (39, 128, 143, 151, 152). Autoantibodies against p53 have been widely associated to CRC due to its highly mutation rate in CRC (33). Several studies revealed the high specificity of p53 autoantibodies in advanced CRC (89-100%), whereas its sensitivity varies from 8.8% to 46.3%, which significantly reduces its diagnostic ability of the disease (138, 143, 153–156). Although most of these studies were focused on advanced stages of the disease, some studies revealed that p53 autoantibodies also possess a high diagnostic ability of the disease at early stages and in individuals with premalignant lesions, with a sensitivity ranging from 10% to 45.2% (130, 138, 157). Interestingly, recent works have supported the diagnostic ability of autoantibodies against other proteoforms of the p53 family (composed of p53, p63, p73, and the different proteoforms of these proteins due to alternative splicing and alternative promoters in the DNA sequence) (126, 130). These proteoforms have been described to regulate (activate or inhibit) different proteins of the p53 protein family, and some of them have been described to be dysregulated and/or mutated in the disease, which might contribute to the production of specific autoantibodies against these proteoforms (125, 158). In this context, autoantibodies against p73, ΔNp73α, and ΔNp73β were found to possess a high diagnostic value for CRC patients (AUC > 65%), with sensitivities and specificities higher than 49% and 86%, respectively, highlighting their ability to discriminate individuals with premalignant lesions from healthy individuals, with AUCs, sensitivities, and specificities higher than 69%, 57%, and 88%, respectively (**Table 5**) (126). In another work from the same groups, the seroreactivity against the different p53 and p63 proteoforms in CRC was investigated. Regarding p53, Δ40p53β, Δ133p53γ, and Δ160p53γ proteoforms showed a high diagnostic ability of CRC patients in comparison to healthy individuals (AUC > 70%), with sensitivities and specificities higher than 67% and 77%, respectively, whereas p53γ, Δ40p53β, Δ40p53γ, Δ133p53γ, and Δ160p53γ proteoforms showed a sensitivity, specificity, and diagnostic ability to discriminate individuals with premalignant lesions from healthy individuals higher than 58%, 51%, and 70%, respectively (**Table 5**). Regarding p63, the TAp63α, TAp63δ, ΔNp63α, and ΔNp63δ proteoforms were able to discriminate both, CRC patients and individuals with premalignant lesions from healthy individuals, with AUCs, sensitivities, and specificities higher than 67%, 42%, and 52%, respectively (**Table 5**) (130). These two works highlighted the significant diagnostic ability of autoantibodies against different p53, p63, and p73 proteoforms, which was higher than that of CEA and autoantibodies against the canonical p53 protein, for both CRC patients and individuals with premalignant lesions, suggesting a potential role of these autoantibodies for the early detection of the disease.

Other autoantibodies have also been identified mainly by ELISA or protein microarrays with diagnostic ability of CRC patients. Among them, 17 have been described in two or more studies (MUC1, c-MYC, Survivin, ANXA4, p62, RPH3AL, NY-CO-16, koc, HDAC5, IMP1, NY-ESO-1, MAGEA3, Cyclin B1, SEC61β, CCCAP, IMPDH2, and GRP78), and they have been described to possesses a sensitivity and specificity ranging from 3-79% and 64-100%, respectively (**Table 5**) (131–150).

Furthermore, plasma samples from CRC patients and healthy individuals were investigated by protein and phage microarrays to identify novel autoantigens with potential diagnosis of the disease. In addition, immunoprecipitation coupled to mass spectrometry analyses were also performed using plasma samples from CRC patients and healthy individuals to investigate novel sources of autoantigens and, thus, to identify novel autoantibodies and their target proteins with potential diagnostic ability of the disease (19, 20, 30, 37–39). From these analyses, ten proteins (PIM1, MAPKAPK3, STK4, SRC, ACVR2B, SULF1, TALDO1, GTF2B, ACTR3, and MT-CO2) and 4 phage-expressed peptides (NHSL1, SREBF2, GRN, and GTF2i) were identified and validated in subsequent works as potential diagnostic autoantigens of CRC, with AUCs higher than 65% (>52% in the case of phage-expressed peptides), and sensitivities and specificities higher than 48% and 62%, respectively. In addition, SPCS2 and RAB2A were also identified in two works as seroreactive to CRC patients although their diagnostic ability of CRC was not estimated by complementary techniques. In addition, ten of these proteins (PIM1, MAPKAPK3, STK4, SRC, SULF1, TALDO1, GTF2B, ACTR3, RAB2A, and MT-CO2) were also found to possess a high diagnostic ability of individuals with premalignant lesions in comparison to healthy individuals, with AUCs, sensitivities, and specificities about 65%, 49%, and 65%, respectively.

Despite that, most of these studies focused on the use of plasma or serum samples from CRC patients (stage I to IV) in comparison to healthy individuals, without taking into account individuals with premalignant lesions. Thus, the inclusion of this group of patients in the validation analyses would be of high interest, as it would highlight those autoantibodies with an early diagnostic capacity of the disease. In addition, the diagnostic capacity of some of these autoantibodies might increase in the early stages, as previously reported for other autoantibodies (19, 20, 38, 126, 130).

However, although most of these described CRC autoantibodies possessed a high specificity (higher than 80%), the individual CRC diagnostic capacity of most of them was very low due to their low sensitivity (lower than 45% in most cases). For this reason, more than 50 panels of autoantibodies combining previously described autoantigens with the best diagnostic abilities of the disease have been proposed as potential diagnostic tools, highlighting the presence of autoantibodies against p53, MUC-1, Cyclin B1, c-MYC, Survivin, PIM1, GTF2B, STK4, and MAPKAPK3 in most of them (151, 152). It is worthy to highlight 15 autoantibody panels including three or more autoantigens with a sensitivity to discriminate CRC patients from healthy individuals higher than 60% (**Table 6**), such as i) STK4 + SULF1 + phage-expressed peptides NHSL1, SREBF2, GRN, GTF2i (AUC: 86%, sensitivity: 82.6%, and specificity: 70%), ii) CHCHD3 + CTTNBP2NL + FKBP4 + MGST3 + THSD7A + TRIM29 (AUC: 99.7%, sensitivity: 96.9%, and specificity: 100%), iii) MAPKAPK3 + ACVR2B + PIM1 (AUC: 85%, sensitivity: 84.4%, and specificity: 71.4%), iv) p53γ + Δ40p53β + Δ133p53γ + TAp63α + TAp63δ + ΔNp63α + ΔNp63δ (AUC: 86.6%, sensitivity: 96.8%, and specificity: 64.6%), v) Tn- + STn + Core3-MUC1, TnMUC4 (sensitivity: 86%, and specificity: 89.3%), and vi) c-MYC + p53 + Cyclin B1 + p62 + koc + IMP1 + Survivin (sensitivity: 88%, and specificity: 88%) (19, 30, 37–39, 128, 130, 132–134, 138, 143, 159).

**Table 6:** Autoantibody panels reported in more than one study for the detection of CRC patients and individuals with colorectal premalignant lesions with high sensitivity and specificity.

Autoantigens panel	AUC (%)	Sensitivity (%)	Specificity (%)	Reference
Diagnosis of CRC patients				
EDIL3+GTF2B+HCK+p53+PIM1+STK4	86.8	65.7	90.0	(37)
EDIL3+GTF2B+HCK+p53+PIM1+STK4 (early stages I and II)	85.9	61.7	90.0	(37)
STK4+SULF1+phage-expressed peptides NHSL1, SREBF2, GRN, GTF2i	86.0	82.6	70.0	(30)
CHCHD3+CTTNBP2NL+FKBP4+MGST3+ THSD7A+TRIM29	99.7	96.9	100	(19)
PIM1+MAPKAPK3+FGFR4	79.7	79.6	84.8	(39)
MAPKAPK3+ACVR2B+PIM1	85.0	84.4	71.4	(128)
GTF2B+MAPKAPK3+PIM1+PKN1+SRC+STK4+SULF1	91.8	76.0	98.6	(38)
p73+ΔNp73α+ΔNp73β	61.5	59.0	90.0	(126)
p53γ+Δ40p53β+Δ133p53γ+TAp63α+TAp63δ+ ΔNp63α+ΔNp63δ	86.6	96.8	64.6	(130)
SLP2+p53+SEC61β+PLSCR1	–	64.0	80.0	(138)
SLP2+p53+SEC61β+PLSCR1 (early stages I and II)	–	66.7	80.0	(138)
p53+c-MYC+Cyclin B1+Cyclin D1+Calnuc	–	65.4	93.7	(133)
koc+p63+IMP3+c-MYC	–	60.9	89.7	(134)
AFP+p53+k-ras+NY-CO-16+RAF1+Annexin	–	75.0	78.0	(143)
Tn-+STn+Core3-MUC1, TnMUC4	–	86.0	89.3	(132)
STn-MUC1, Tn-MUC4-1-Tn-MUC4-5	–	79.3	95.1	(132)
c-MYC+p53+Cyclin B1+p62+koc+IMP1+Survivin	–	88.0	88.0	(159)
Diagnosis of individuals with premalignant lesions				
CTTNBP2NL+FKBP4+TALDO1	88.9	83.3	71.4	(19)
MAPKAPK3+PIM1+PKN1+SRC+STK4+SULF1	83.0	84.0	75.7	(38)
p73+ΔNp73α+ΔNp73β	79.7	73.3	93.3	(126)
p53γ+Δ40p53β+Δ40p53γ+Δ133p53γ+Δ160p53γ+TAp63α+TAp63δ+ΔNp63α+ΔNp63δ	91.3	77.4	91.7	(130)

Additionally, four panels of autoantibodies have been also described to discriminate individuals with premalignant lesions from healthy individuals with a high AUC (>83%), sensitivity (>77%), and specificity (>75%), suggesting that these panels could be useful for early and preventive diagnosis of CRC (**Table 6**).

Finally, although there are few works performing a follow-up of CRC patients to evaluate the prognostic ability of autoantibodies, it has been postulated that some of them have CRC prognostic capacity. Autoantibodies against ADAM10 were demonstrated to possess a high diagnostic ability of CRC (AUC > 65%), whereas the follow-up of stage III CRC patients from 2 to 73 months revealed the association of high ADAM10 plasma autoantibodies with an increased recurrence-free survival rate (from 23 to 55 months) (160). In addition, TOPO48 autoantibodies were also shown to have high diagnostic capacity in individuals with premalignant lesions (AUC 83.5%), and the 3 to 36 months follow-up of CRC patients showed an increased overall survival rate of patients when TOPO48 plasma autoantibodies at early stages were high (161). In contrast, although high p53 autoantibody levels have been associated with adenocarcinoma and invasive carcinomas, no differences based on clinical stage, overall survival, or disease-free survival were found associated to p53 autoantibodies during the follow-up of CRC patients. Additionally, high p53 autoantibody levels in plasma have been considered a risk factor of CRC recurrence (157, 162, 163). In this sense, some autoantibodies have been also postulated as associated to the progression of the disease, with high autoantibody levels against VTI2 and p53 associated to CRC metastasis, or against GRP78 and SPAG9 to early stages of the disease (15, 149, 164).

### 4.4 Autoantibodies in prostate cancer

Among men, prostate cancer (PC) is one of the most prevalent cancer types. More prevalent in developed countries, its incidence is also higher among men of African descent. The incidence rates increase with age, with most cases diagnosed in men over 65 years old. For its diagnosis and management, the detection of the prostate specific antigen (PSA) offers an interesting alternative as an initial screening method followed by confirmatory techniques (59). However, since patients with benign prostatic hyperplasia also show elevated PSA levels, PSA detection produces an elevated number of false positives, raising concerns about overdiagnosis and overtreatment. Furthermore, it is worth noting that not all PC patients show elevated PSA levels, limiting even more its use as a single diagnostic marker. Such low specificity for PC detection indicates the need for a better, less stressful alternative to be described for PC detection, since overtreatment of the disease has been widely described (165). In this context, the U.S. Preventive Services Task Force (USPSTF) recommended in 2020 against PSA screening. This recommendation was based on negative results, as well as evidence of potential harms arising from PSA testing. These harms include overdiagnosis and treatment of small, benign-appearing cancers that are unlikely to spread or lead to death. Therefore, due to this low detection specificity and PSA controversy worldwide, the measurement of PSA levels for PC detection is accompanied by complimentary detection techniques such as digital rectal examination, trans-rectal ultrasonography, or multiparametric magnetic resonance imaging (166). Additionally, numerous tests are commercially available for the stratification of patients according to the disease’s outcome (167). Nowadays, there are numerous studies focused on the development of diagnostic platforms that can increase PSA PC specificity, demonstrating the combined measurement of its levels coupled to the detection of other markers possess higher diagnostic ability of the disease.

As with other cancer types, PC patients have been shown to develop TAAbs against numerous proteins able to discriminate between patients and healthy controls (**Table 7**). Moreover, the presence of these antibodies has been used as clinical confirmation of the disease in diagnosed patients and numerous studies have detected potential autoantibodies that could aid in PC diagnosis and patient stratification (167, 172–178). However, not all of the described autoantibodies have been validated in two or more independent studies, so further analysis is required. In a study involving protein microarrays, autoantibodies against AMACR, a tissue biomarker for PC, was found to discriminate between PC patients and healthy controls with 71.8% sensitivity and 61.6% specificity (168). Another study reported an AUC of 74% when detecting AMACR autoantibodies in PC patients, but no sensitivity or specificity was reported (179). However, no information regarding its selectivity against other cancer types is specified in a humoral immune response scenario, so AMACR autoantibody detection currently resides more as a confirmation marker instead of initial PC diagnosis. Moreover, autoantibodies against HIP1, a protein up-regulated in PC when comparing its levels to benign prostatic epithelia and overexpressed in advanced PC (180), could discriminate between patients and controls with up to 64% specificity and 88% sensitivity (169). However, to our knowledge, only one study has reported their usefulness in PC diagnosis, and further evaluation should be carried. Moreover, in this study the same samples tested for the presence of HIP1 autoantibodies were tested for AMACR autoantibodies, and values of 67% specificity and 64% sensitivity were obtained. When combining the detection of both autoantibodies, the specificity increased to 97%, suggesting that the combined detection of both autoantibodies, together with PSA, could serve as a better diagnostic tool for PC than when analyzed individually. It is interesting to note that some autoantibodies have been described in PC patients in more than one study, but their diagnostic potential remains to be clarified. Such are the examples of autoantibodies against NY-ESO-1 (167, 181) and GAG-HERV-K (167, 182, 183). On the other hand, another report centered on previous studies in which cyclin B1 autoantibodies were found in PC patients and its inclusion in autoantibody panels could detect the pathology (135, 159, 184) concluded that the combined presence of autoantibodies against cyclin B1 and elevated PSA levels could identify 65.7% of patients with early PC (170). This same study also concluded that the combined presence of autoantibodies against cyclin B1, Survivin, p53, RalA, DFS70/LEDGFp75, MDM2, and NPM1 could discriminate between PC patients and healthy controls with 80.5% sensitivity and 91% specificity, although follow-up studies are needed to verify its diagnostic value.

**Table 7:** Autoantibodies reported for the detection of PC patients.

Autoantigen(s) detection	AUC (%)	Sensitivity (%)	Specificity (%)	Reference
PC vs healthy controls				
AMACR	66.3	71.8	61.6	(168)
AMACR	–	64	67	(169)
HIP1	–	88	64	(169)
AMACR + HIP1	–	55	97	(169)
Cyclin B1 + Survivin + p53 + RalA + DFS70/LEDGFp75 + MDM2 + NPM1	94.2	80.5	91	(170)
PC vs benign prostate hyperplasia				
TARDBP + TLN1 + PARK7 + LEDGD + CALD1	95	95	80	(171)

Regarding the distinction between patients with PC and patients with benign prostate hyperplasia, autoantibodies against cyclin B1 were found present in 31.0% of sera from patients with pancreatic cancer while only in 4.8% of patients with benign prostatic hyperplasia (170). Other microarray studies showed that a signature comprised of autoantibodies against TARDBP, TLN1, PARK7, LEDGD, and CALD1 could distinguish between groups with 95% sensitivity and 80% specificity (AUC = 95%), making them an interesting choice for patient stratification (171) and highlighting, once more, the importance of multiple autoantibody detection for a better discrimination of individuals. Further studies developed a sensor for PC diagnosis that could detect autoantibodies against four of the previous proteins (TARDBP, PARK7, TLNQ, and CALD1) and total PSA and free-PSA (AUC = 91.6%) (185). Another study employing microarrays containing more than 37,000 recombinant human proteins identified 174 autoantibodies found only in sera from PC in comparison to healthy individuals and individuals with benign disease. Different autoantibody profiles were validated as capable of discriminating between cancer and benign patients, with TTLL12 autoantibodies as the most effective (186).

Autoantibody detection has also been proven as an interesting alternative to detect PC patients that show normal PSA levels. Autoantibodies against cyclin B1, an antigen found to be very reactive in this type of cancer, were found in 31% of evaluated patients, and 29% of the patients with normal PSA levels had these autoantibodies (170). Therefore, autoantibodies in PC seem to be independent of PSA levels, making them an interesting alternative to detect patients that might be overlooked due to their normal PSA levels. Therefore, the combined detection of autoantibodies and PSA levels would then allow for an ampler identification of PC individuals.

Finally, regarding the prognostic ability of PC autoantibodies, individual autoantibodies against GRP78 (187), fetuin-A (175), and GAG-HERV-K (183) have been demonstrated to predict a more aggressive outcome of PC. High throughput methods based on peptide microarrays have also identified different signatures of autoantibody combinations that vary according to disease stage, making them an interesting alternative for the study of prognosis of PC (172).

## 5 Leveraging the humoral immune response to discover potential druggable therapeutic targets in cancer patients

The immune system, being an extremely sensitive detector for identifying altered self-proteins during the neoplastic process, can aid in identifying plasma membrane proteins that could serve as therapeutic targets. Indeed, several reports have highlighted the identification of such potential therapeutic targets.

Two protein families, kinases and phosphatases, have been reported as target of autoantibodies with potential therapeutic value. Kinases are common targets for therapeutic drugs, and the presence of autoantibodies against kinases in CRC, hematological cancers, non-small cell LC (30, 128, 188–191), among others, suggests that at least part of the humoral immune response occurs against cancer targets druggable proteins. For instance, FGFR4, a target of autoantibodies with significant diagnostic ability for CRC, has been reported to be druggable using CRC cells with either broad-spectrum kinase inhibitors like TKI-258 or more selective FGFR inhibitors like PD173074, or specific antibody inhibitors in phase III (128, 192). Subsequent studies have demonstrated the potential of FGFR4 as a therapeutic target in BC and hepatocellular cancer (193, 194). Given that FGFR4 inhibitors are currently in early-stage clinical trials for treatment, this protein, a target of autoantibodies, is of interest as a druggable target in BC, CRC, and hepatocellular cancers (195). In addition to FGFR4, other kinases such as IRAK4, CKMT1B, PKN1, MAPKAPK3, Pim1, and STK4 in CRC, ALK in LC, CSNK1A1L and Her2/Neu in BC, and ECPKA in Non-Hodgkin’s lymphoma, breast, colon, and most highly incident cancers have been observed both as target of autoantibodies with diagnostic ability and as potential targets for therapy (30, 128, 190, 196, 197). In this sense, PIM1, STK4, CSNK1A1L, ALK, and ECPKA, are currently under investigation as therapeutic targets with specific inhibitors under development, or commercialized drugs have been developed, as for ALK (28, 198, 199). On the other hand, tyrosine kinase signaling is switched off by tyrosine-phosphatases. The phosphotyrosine phosphatase receptor (PTPR) protein family, which comprises 21 members, is frequently altered in cancer, with some members exhibiting oncogenic and tumor suppressor features (200, 201). Although oncogenic PTPRs are attractive molecules for the development of targeted therapies, the development of PTPR inhibitors remains challenging due to the high level of conservation in the active site of tumor suppressor and oncogenic PTPRs (201). Notably, among the 21 members of the family, PTPRN and PTPRA have been reported to induce a humoral immune response in CRC and BC patients, respectively. These proteins are not only included in interesting autoantibody panels for diagnosis but also proposed as therapeutic targets in both cancers (196, 202). In this regard, PTPRN overexpression has been reported in advanced CRC and metastatic CRC cells to the liver. The fact that the depletion of this protein abrogates the liver colonization properties of CRC cells makes PTPRN an interesting therapeutic target for metastatic CRC (202).

Finally, with the aim to enhance tumor autoantibody potential to develop specific antigenic/antibody repertoires as cancer vaccines based on the observation that mutated tumor suppressors such as p53 served as primers for T cell–mediated and antibody driven responses (203), several therapeutic pan-cancer vaccines based on TAAs have been evaluated in different phases of clinical trials addressing different cancer malignancies, as NY-ESO-1, Survivin, or MAGEA1 in different cancer types (204, 205). In BC, the majority of TAAs studied as BC vaccines are the HER2 protein and other HER2-derived peptides (204), MUC-1 (206), and a number of cancer testis antigens including KK-LC-1, NY-ESO-1, and MAGEA1 (204), with NY-ESO-1, and MAGEA1 as major TAAs also in LC (99, 105, 106). In LC, immunotherapy has emerged as a standard of care for stage III-IV non-small cell LC (207). Regarding vaccines based on specific LC TAAs, besides NY-ESO-1 and MAGEA1 analyses, MAGEA4 has been analyzed as a universal immunoprevention cancer vaccine (208), and Survivin has been the target of long synthetic peptide (205). In PC, several vaccines based on autoantigens target of autoantibodies have also undergone clinical trials. In this sense, although several vaccines composed of peptides against TAAs highly expressed in PC have been clinically analyzed in phase I and phase II studies, it has been preferred the analysis of individualized polypeptide vaccines (209). Regarding vaccines based on specific PC TAAs target of autoantibodies, although PC vaccines should be particularly promising treatment options because PC develops slower than most cancers (209), only the analyses of NY-ESO-1 or Survivin have been also reported as cancer vaccines based on TAAs target of autoantibodies, with no other studies related to the TAAs discussed here as more specific of PC. Finally, in CRC several TAAs target of autoantibodies have been widely explored in clinical trials as cancer vaccines as CEA, and MAGE (210), together with other pan-explored TAAs in different cancers as MUC1, EGFR, or Survivin (211).

However, till now these approaches have shown little correlation with favorable clinical outcomes (212). This suggests more personalized treatments should be tried based on the individual autoantibody profiles of the patients, or by using those TAAs appearing in multiple reports as cancer-type specific vaccines, besides current therapeutic strategies. Alternatively, these results could also importantly suggest using the humoral immune response in cancer patients to identify those target proteins of cancer autoantibodies prone to become cancer-specific therapeutic targets for development of more personalized therapies or for the development of autoepitope immunotherapies approaches for personalized therapy. Therefore, there is still room for the development of cancer specific vaccines based on TAAs target of autoantibodies.

## 6 Conclusions/emergent diagnostic platforms and future prospects

Although the identification of TAAs is yet to be completed by combining the various approaches described here, especially for the most prevalent cancers (BC, LC, CRC, and PC), cancer autoantigens are currently a viable strategy for cancer diagnosis and prognosis, particularly at very early stages of the disease. Moreover, an important point to have also in mind is that several autoantibodies exist in different cancer malignancies, as p53, NY-ESO-1, MUC1, MAGEA1, or Survivin, and thus autoantibodies and their target TAAs should be tested using plasma or serum samples from a battery of different cancer malignancies to determine their exact specificity. In this sense, few studies demonstrate the specificity of different TAAs using samples from different malignancies (20, 38), and thus demonstrating the specificity of the TAAs to a specific cancer type. The immune system’s ability to detect minor protein alterations makes the strategy of monitoring the humoral immune response of individuals at high risk of developing cancer very promising. In this sense, it should be recommendable to include in the same diagnostic/prognostic panel, promiscuous cancer specific autoantibodies as p53, MUC1, NY-ESO-1, MAGEA1, and Survivin, among others, together with specific autoantibodies of different cancer types as HER2 or BRCA2 in BC, SOX2, or GBU4-5 in LC, AMACR, or PARK7 in PC, and CEA, PIM1, or GTF2B in CRC to be able to detect not only cancer but its exact localization. Thus, this strategy could be used for the classification of cancer patients and for defining potential individual targets of intervention as a first step towards personalized medicine. The minimal invasiveness required for obtaining serum or plasma should facilitate the implementation of cancer autoantibodies in routine health analyses.

This is of particular relevance when measured through novel multiplexed methodologies that are POC-like and compatible with clinical settings, as they require low volumes of biological samples and are not time-consuming (20, 38, 41, 42). These latter methodologies as SIMOA or biosensing approaches as electrochemical bioplatforms are at the forefront of modern detection techniques. Their high selectivity and sensitivity, ease of use and low cost together with their fast response time and feasibility to operate at the multiplexed and multiomics level either in centralized or field settings owed the possibility to implement them in clinics for the analyses of autoantibody panels. Indeed, in this sense, biosensing approaches for autoantibody detection in chronic diseases have been reported (42, 213, 214), including CRC (20, 38, 41). Therefore, it is expected that in the following years multiplexed biosensing approaches will be implemented for population screening and/or clinical routine to detect different cancer types with just a blood test. This would allow to identify patients at early clinical stages to improve cancer patient survival rates, resulting at the same time in significant savings for National Health Systems, as it is considerably cheaper to treat cancer patients at early and curable stages than at advanced stages where patient survival is compromised. This is in contrast to other invasive or costly screening methods, such as imaging approaches as mammography, or colonoscopy -among others-, which cannot be implemented for global population screening.

Finally, the ultimate endorsement for the use of autoantibodies for cancer diagnosis and monitoring will come from clinical trials. In this regard, there are currently in the market kits based on the identification of LC autoantibodies for early detection of LC (104, 215), and one recently approved in the US for the detection of BC -the Videssa Breast test- through the detection of serum protein biomarkers and TAAbs with >98% accuracy in women with suspicious imaging findings (81, 83). Therefore, it is expected the arrival to the market of other tests based on autoantibodies or in combination with other multiomic markers for the detection of the most prevalent cancers. Moreover, although more research is still needed in these areas, completing the cancer autoantibodyome, irrespective of diagnosis, monitoring, or patient prognosis, should also aid in identifying therapeutic targets for intervention and therapy monitoring.

## References

[1] Chen VE Greenberger BA Taylor JM Edelman MJ Lu B. The underappreciated role of the humoral immune system and B cells in tumorigenesis and cancer therapeutics: A review. Int J Radiat Oncol Biol Phys. (2020) 108:38–45. doi: 10.1016/j.ijrobp.2020.03.022 32251756

[2] Reuschenbach M von Knebel Doeberitz M Wentzensen N. A systematic review of humoral immune responses against tumor antigens. Cancer Immunol Immunother. (2009) 58:1535–44. doi: 10.1007/s00262-009-0733-4 PMC2782676 19562338

[3] Zaenker P Gray ES Ziman MR. Autoantibody production in cancer–the humoral immune response toward autologous antigens in cancer patients. Autoimmun Rev. (2016) 15:477–83. doi: 10.1016/j.autrev.2016.01.017 26827909

[4] Da Gama Duarte J Peyper JM. Blackburn JM. B cells and antibody production in melanoma. Mamm Genome. (2018) 29:790–805. doi: 10.1007/s00335-018-9778-z 30178304

[5] Patel AJ Richter A Drayson MT Middleton GW. The role of B lymphocytes in the immuno-biology of non-small-cell lung cancer. Cancer Immunol Immunother. (2020) 69:325–42. doi: 10.1007/s00262-019-02461-2 PMC7044257 31901949

[6] Gandhi SJ Minn AJ Vonderheide RH Wherry EJ Hahn SM Maity A. Awakening the immune system with radiation: optimal dose and fractionation. Cancer Lett. (2015) 368:185–90. doi: 10.1016/j.canlet.2015.03.024 25799953

[7] Golden EB Chhabra A Chachoua A Adams S Donach M Fenton-Kerimian M. Local radiotherapy and granulocyte-macrophage colony-stimulating factor to generate abscopal responses in patients with metastatic solid tumours: A proof-of-principle trial. Lancet Oncol. (2015) 16:795–803. doi: 10.1016/S1470-2045(15)00054-6 26095785

[8] Anderson KS LaBaer J. The sentinel within: exploiting the immune system for cancer biomarkers. J Proteome Res. (2005) 4:1123–33. doi: 10.1021/pr0500814 PMC2522321 16083262

[9] Murphy MA O’Leary JJ Cahill DJ. Assessment of the humoral immune response to cancer. J Proteomics. (2012) 75:4573–9. doi: 10.1016/j.jprot.2012.01.021 22300580

[10] Barderas R Villar-Vazquez R Fernandez-Acenero MJ Babel I Pelaez-Garcia A Torres S. Sporadic colon cancer murine models demonstrate the value of autoantibody detection for preclinical cancer diagnosis. Sci Rep. (2013) 3:2938. doi: 10.1038/srep02938 24126910 PMC3796738

[11] Kobold S Lutkens T Cao Y Bokemeyer C Atanackovic D. Autoantibodies against tumor-related antigens: incidence and biologic significance. Hum Immunol. (2010) 71:643–51. doi: 10.1016/j.humimm.2010.03.015 20433885

[12] Carey TE Takahashi T Resnick LA Oettgen HF Old LJ. Cell surface antigens of human Malignant melanoma: mixed hemadsorption assays for humoral immunity to cultured autologous melanoma cells. Proc Natl Acad Sci U.S.A. (1976) 73:3278–82. doi: 10.1073/pnas.73.9.3278 PMC431008 1067619

[13] Shiku H Takahashi T Oettgen HF. Cell surface antigens of human Malignant melanoma. Ii. Serological typing with immune adherence assays and definition of two new surface antigens. J Exp Med. (1976) 144:873–81. doi: 10.1084/jem.144.4.873 PMC2190439 978138

[14] Shiku H Takahashi T Resnick LA Oettgen HF Old LJ. Cell surface antigens of human Malignant melanoma. Iii. Recognition of autoantibodies with unusual characteristics. J Exp Med. (1977) 145:784–9. doi: 10.1084/jem.145.3.784 PMC2180718 233917

[15] Gonzalez-Gonzalez M Sayagues JM Munoz-Bellvis L Pedreira CE de Campos MLR Garcia J. Tracking the antibody immunome in sporadic colorectal cancer by using antigen self-assembled protein arrays. Cancers (Basel). (2021) 13(11):2718. doi: 10.3390/cancers13112718 34072782 PMC8198956

[16] Song L Song M Camargo MC Van Duine J Williams S Chung Y. Identification of anti-Epstein-barr virus (Ebv) antibody signature in Ebv-associated gastric carcinoma. Gastric Cancer. (2021) 24:858–67. doi: 10.1007/s10120-021-01170-z PMC8206016 33661412

[17] Ren J Wang H Wei C Yang X Yu X. Development of a protein microarray for profiling circulating autoantibodies in human diseases. Proteomics Clin Appl. (2022) 16(6):e2100132. doi: 10.1002/prca.202100132 36006834

[18] Gahoi N Syed P Choudhary S Epari S Moiyadi A Varma SG. A protein microarray-based investigation of cerebrospinal fluid reveals distinct autoantibody signature in low and high-grade gliomas. Front Oncol. (2020) 10:543947. doi: 10.3389/fonc.2020.543947 33415070 PMC7784397

[19] Garranzo-Asensio M San Segundo-Acosta P Poves C Fernandez-Acenero MJ Martinez-Useros J Montero-Calle A. Identification of tumor-associated antigens with diagnostic ability of colorectal cancer by in-depth immunomic and seroproteomic analysis. J Proteomics. (2020) 214:103635. doi: 10.1016/j.jprot.2020.103635 31918032

[20] Montero-Calle A Aranguren-Abeigon I Garranzo-Asensio M Povés C Fernandez-Acenero MJ Martinez-Useros J. Multiplexed biosensing diagnostic platforms detecting autoantibodies to tumor-associated antigens from exosomes released by crc cells and tissue samples showed high diagnostic ability for colorectal cancer. Engineering. (2021) 7:1393–412. doi: 10.1016/j.eng.2021.04.026

[21] Ayoglu B Haggmark A Khademi M Olsson T Uhlen M Schwenk JM. Autoantibody profiling in multiple sclerosis using arrays of human protein fragments. Mol Cell Proteomics. (2013) 12:2657–72. doi: 10.1074/mcp.M112.026757 PMC3769337 23732997

[22] Gupta S Mukherjee S Syed P Pandala NG Choudhary S Singh VA. Evaluation of autoantibody signatures in meningioma patients using human proteome arrays. Oncotarget. (2017) 8:58443–56. doi: 10.18632/oncotarget.16997 PMC5601665 28938569

[23] San Segundo-Acosta P Montero-Calle A Jernbom-Falk A Alonso-Navarro M Pin E Andersson E. Multiomics profiling of Alzheimer’s disease serum for the identification of autoantibody biomarkers. J Proteome Res. (2021) 20:5115–30. doi: 10.1021/acs.jproteome.1c00630 34628858

[24] Ray S Reddy PJ Jain R Gollapalli K Moiyadi A Srivastava S. Proteomic technologies for the identification of disease biomarkers in serum: advances and challenges ahead. Proteomics. (2011) 11:2139–61. doi: 10.1002/pmic.201000460 21548090

[25] Pan J Song G Chen D Li Y Liu S Hu S. Identification of serological biomarkers for early diagnosis of lung cancer using a protein array-based approach. Mol Cell Proteomics. (2017) 16:2069–78. doi: 10.1074/mcp.RA117.000212 PMC5724172 29021294

[26] Banerjee A Ray A Barpanda A Dash A Gupta I Nissa MU. Evaluation of autoantibody signatures in pituitary adenoma patients using human proteome arrays. Proteomics Clin Appl. (2022) 16(6):e2100111. doi: 10.1002/prca.202100111 35939377

[27] Ling HZ Xu SZ Leng RX Wu J Pan HF Fan YG. Discovery of new serum biomarker panels for systemic lupus erythematosus diagnosis. Rheumatol (Oxford). (2020) 59:1416–25. doi: 10.1093/rheumatology/kez634 31899518

[28] Thul PJ Akesson L Wiking M Mahdessian D Geladaki A Ait Blal H. A subcellular map of the human proteome. Science. (2017) 356(6340):eaal3321. doi: 10.1126/science.aal3321 28495876

[29] Uhlen M Fagerberg L Hallstrom BM Lindskog C Oksvold P Mardinoglu A. Proteomics. Tissue-based map of the human proteome. Science. (2015) 347:1260419. doi: 10.1126/science.1260419 25613900

[30] Babel I Barderas R Diaz-Uriarte R Moreno V Suarez A Fernandez-Acenero MJ. Identification of mst1/stk4 and sulf1 proteins as autoantibody targets for the diagnosis of colorectal cancer by using phage microarrays. Mol Cell Proteomics. (2011) 10:M110 001784. doi: 10.1074/mcp.M110.001784 PMC3047148 21228115

[31] San Segundo-Acosta P Montero-Calle A Fuentes M Rabano A Villalba M Barderas R. Identification of Alzheimer’s disease autoantibodies and their target biomarkers by phage microarrays. J Proteome Res. (2019) 18:2940–53. doi: 10.1021/acs.jproteome.9b00258 31136180

[32] Chatterjee M Hurley LC Tainsky MA. Paraneoplastic antigens as biomarkers for early diagnosis of ovarian cancer. Gynecol Oncol Rep. (2017) 21:37–44. doi: 10.1016/j.gore.2017.06.006 28653032 PMC5476453

[33] Katchman BA Barderas R Alam R Chowell D Field MS Esserman LJ. Proteomic mapping of P53 immunogenicity in pancreatic, ovarian, and breast cancers. Proteomics Clin Appl. (2016) 10:720–31. doi: 10.1002/prca.201500096 PMC5553208 27121307

[34] Lin L Zheng J Yu Q Chen W Xing J Chen C. High throughput and accurate serum proteome profiling by integrated sample preparation technology and single-run data independent mass spectrometry analysis. J Proteomics. (2018) 174:9–16. doi: 10.1016/j.jprot.2017.12.014 29278786

[35] Rezaei M Nikeghbalian S Mojtahedi Z Ghaderi A. Identification of antibody reactive proteins in pancreatic cancer using 2d immunoblotting and mass spectrometry. Oncol Rep. (2018) 39:2413–21. doi: 10.3892/or.2018.6285 29498409

[36] Garranzo-Asensio M Guzman-Aranguez A Poves C Fernandez-Acenero MJ Montero-Calle A Ceron MA. The specific seroreactivity to np73 isoforms shows higher diagnostic ability in colorectal cancer patients than the canonical P73 protein. Sci Rep. (2019) 9:13547. doi: 10.1038/s41598-019-49960-x 31537884 PMC6753153

[37] Villar-Vazquez R Padilla G Fernandez-Acenero MJ Suarez A Fuente E Pastor C. Development of a novel multiplex beads-based assay for autoantibody detection for colorectal cancer diagnosis. Proteomics. (2016) 16:1280–90. doi: 10.1002/pmic.201500413 26915739

[38] Garranzo-Asensio M Guzman-Aranguez A Povedano E Ruiz-Valdepenas Montiel V Poves C Fernandez-Acenero MJ. Multiplexed monitoring of a novel autoantibody diagnostic signature of colorectal cancer using halotag technology-based electrochemical immunosensing platform. Theranostics. (2020) 10:3022–34. doi: 10.7150/thno.42507 PMC7053203 32194852

[39] Barderas R Babel I Diaz-Uriarte R Moreno V Suarez A Bonilla F. An optimized predictor panel for colorectal cancer diagnosis based on the combination of tumor-associated antigens obtained from protein and phage microarrays. J Proteomics. (2012) 75:4647–55. doi: 10.1016/j.jprot.2012.03.004 22465712

[40] Montero-Calle A San Segundo-Acosta P Garranzo-Asensio M Rabano A Barderas R. The molecular misreading of app and ubb induces a humoral immune response in Alzheimer’s disease patients with diagnostic ability. Mol Neurobiol. (2020) 57:1009–20. doi: 10.1007/s12035-019-01809-0 31654319

[41] Garranzo-Asensio M Guzman-Aranguez A Poves C Fernandez-Acenero MJ Torrente-Rodriguez RM Ruiz-Valdepenas Montiel V. Toward liquid biopsy: determination of the humoral immune response in cancer patients using halotag fusion protein-modified electrochemical bioplatforms. Anal Chem. (2016) 88:12339–45. doi: 10.1021/acs.analchem.6b03526 28193070

[42] Campuzano S Barderas R Yanez-Sedeno P Pingarron JM. Electrochemical biosensing to assist multiomics analysis in precision medicine. Curr Opin Electrochemistry. (2021) 28:100703. doi: 10.1016/j.coelec.2021.100703

[43] Van der Schouw YT Verbeek AL Ruijs JH. Roc curves for the initial assessment of new diagnostic tests. Fam Pract. (1992) 9:506–11. doi: 10.1093/fampra/9.4.506 1490547

[44] Hajian-Tilaki K. Receiver operating characteristic (Roc) curve analysis for medical diagnostic test evaluation. Caspian J Intern Med. (2013) 4:627–35. PMC3755824 24009950

[45] Baumgarth N. The double life of a B-1 cell: self-reactivity selects for protective effector functions. Nat Rev Immunol. (2011) 11:34–46. doi: 10.1038/nri2901 21151033

[46] Elkon K Casali P. Nature and functions of autoantibodies. Nat Clin Pract Rheumatol. (2008) 4:491–8. doi: 10.1038/ncprheum0895 PMC2703183 18756274

[47] Nagele EP Han M Acharya NK DeMarshall C Kosciuk MC Nagele RG. Natural igg autoantibodies are abundant and ubiquitous in human sera, and their number is influenced by age, gender, and disease. PloS One. (2013) 8:e60726. doi: 10.1371/journal.pone.0060726 23589757 PMC3617628

[48] Islam Roney MMS Lanagan MC Sheng Y Gough MM Snell C Nguyen NT. Isotypic analysis of anti-P53 serum autoantibodies and P53 protein tissue phenotypes in colorectal cancer. Hum Pathol. (2022) 128:1–10. doi: 10.1016/j.humpath.2022.06.017 35750247

[49] Suppiah A Greenman J. Clinical utility of anti-P53 auto-antibody: systematic review and focus on colorectal cancer. World J Gastroenterol. (2013) 19:4651–70. doi: 10.3748/wjg.v19.i29.4651 PMC3732838 23922463

[50] Soussi T. P53 antibodies in the sera of patients with various types of cancer: A review. Cancer Res. (2000) 60:1777–88. 10766157

[51] Sung H Ferlay J Siegel RL Laversanne M Soerjomataram I Jemal A. Global cancer statistics 2020: globocan estimates of incidence and mortality worldwide for 36 cancers in 185 countries. CA Cancer J Clin. (2021) 71:209–49. doi: 10.3322/caac.21660 33538338

[52] Hong R Xu B. Breast cancer: an up-to-date review and future perspectives. Cancer Commun (Lond). (2022) 42:913–36. doi: 10.1002/cac2.12358 PMC9558690 36074908

[53] Harbeck N Penault-Llorca F Cortes J Gnant M Houssami N Poortmans P. Breast cancer. Nat Rev Dis Primers. (2019) 5(1):66. doi: 10.1038/s41572-019-0111-2 31548545

[54] Lukasiewicz S Czeczelewski M Forma A Baj J Sitarz R Stanislawek A. Breast cancer-epidemiology, risk factors, classification, prognostic markers, and current treatment strategies-an updated review. Cancers (Basel). (2021) 13(17):4287. doi: 10.3390/cancers13174287 34503097 PMC8428369

[55] Yang R Han Y Yi W Long Q. Autoantibodies as biomarkers for breast cancer diagnosis and prognosis. Front Immunol. (2022) 13:1035402. doi: 10.3389/fimmu.2022.1035402 36451832 PMC9701846

[56] Qiu J Keyser B Lin ZT Wu T. Autoantibodies as potential biomarkers in breast cancer. Biosensors (Basel). (2018) 8(3):67. doi: 10.3390/bios8030067 30011807 PMC6163859

[57] Rauf F Anderson KS LaBaer J. Autoantibodies in early detection of breast cancer. Cancer Epidemiol Biomarkers Prev. (2020) 29:2475–85. doi: 10.1158/1055-9965.EPI-20-0331 PMC7710604 32994341

[58] Chapman C Murray A Chakrabarti J Thorpe A Woolston C Sahin U. Autoantibodies in breast cancer: their use as an aid to early diagnosis. Ann Oncol. (2007) 18:868–73. doi: 10.1093/annonc/mdm007 17347129

[59] Bray F Ren JS Masuyer E Ferlay J. Global estimates of cancer prevalence for 27 sites in the adult population in 2008. Int J Cancer. (2013) 132:1133–45. doi: 10.1002/ijc.27711 22752881

[60] Sumazaki M Ogata H Nabeya Y Kuwajima A Hiwasa T Shimada H. Multipanel assay of 17 tumor-associated antibodies for serological detection of stage 0/I breast cancer. Cancer Sci. (2021) 112:1955–62. doi: 10.1111/cas.14860 PMC8088936 33605508

[61] Lu H Ladd J Feng Z Wu M Goodell V Pitteri SJ. Evaluation of known oncoantibodies, her2, P53, and cyclin B1, in prediagnostic breast cancer sera. Cancer Prev Res (Phila). (2012) 5:1036–43. doi: 10.1158/1940-6207.CAPR-11-0558 PMC3790582 22715141

[62] Schummer M Thorpe J Giraldez MD Bergan L Tewari M Urban N. Evaluating serum markers for hormone receptor-negative breast cancer. PloS One. (2015) 10:e0142911. doi: 10.1371/journal.pone.0142911 26565788 PMC4643893

[63] Mudenda B Green JA Green B Jenkins JR Robertson L Tarunina M. The relationship between serum P53 autoantibodies and characteristics of human breast cancer. Br J Cancer. (1994) 69:1115–9. doi: 10.1038/bjc.1994.219 PMC1969453 8198980

[64] Blixt O Bueti D Burford B Allen D Julien S Hollingsworth M. Autoantibodies to aberrantly glycosylated muc1 in early stage breast cancer are associated with a better prognosis. Breast Cancer Res. (2011) 13:R25. doi: 10.1186/bcr2841 21385452 PMC3219186

[65] von Mensdorff-Pouilly S Gourevitch MM Kenemans P Verstraeten AA Litvinov SV van Kamp GJ. Humoral immune response to polymorphic epithelial mucin (Muc-1) in patients with benign and Malignant breast tumours. Eur J Cancer. (1996) 32A:1325–31. doi: 10.1016/0959-8049(96)00048-2 8869094

[66] Liu W Li Y Wang B Dai L Qian W Zhang JY. Autoimmune response to igf2 mrna-binding protein 2 (Imp2/P62) in breast cancer. Scand J Immunol. (2015) 81:502–7. doi: 10.1111/sji.12285 PMC4431935 25721883

[67] Desmetz C Bibeau F Boissiere F Bellet V Rouanet P Maudelonde T. Proteomics-based identification of hsp60 as a tumor-associated antigen in early stage breast cancer and ductal carcinoma in situ. J Proteome Res. (2008) 7:3830–7. doi: 10.1021/pr800130d 18683965

[68] He Y Wu Y Mou Z Li W Zou L Fu T. Proteomics-based identification of hsp60 as a tumor-associated antigen in colorectal cancer. Proteomics Clin Appl. (2007) 1:336–42. doi: 10.1002/prca.200600718 21136683

[69] Disis ML Pupa SM Gralow JR Dittadi R Menard S Cheever MA. High-titer her-2/neu protein-specific antibody can be detected in patients with early-stage breast cancer. J Clin Oncol. (1997) 15:3363–7. doi: 10.1200/JCO.1997.15.11.3363 9363867

[70] Goodell V Waisman J Salazar LG de la Rosa C Link J Coveler AL. Level of her-2/neu protein expression in breast cancer may affect the development of endogenous her-2/neu-specific immunity. Mol Cancer Ther. (2008) 7:449–54. doi: 10.1158/1535-7163.MCT-07-0386 18319334

[71] Bassaro L Russell SJ Pastwa E Somiari SA Somiari RI. Screening for multiple autoantibodies in plasma of patients with breast cancer. Cancer Genomics Proteomics. (2017) 14:427–35. doi: 10.21873/cgp.20052 PMC6070323 29109092

[72] Yagihashi A Ohmura T Asanuma K Kobayashi D Tsuji N Torigoe T. Detection of autoantibodies to survivin and livin in sera from patients with breast cancer. Clin Chim Acta. (2005) 362:125–30. doi: 10.1016/j.cccn.2005.06.009 16026775

[73] Xiu Y Sun B Jiang Y Wang A Liu L Liu Y. Diagnostic value of the survivin autoantibody in four types of Malignancies. Genet Test Mol Biomarkers. (2018) 22:384–9. doi: 10.1089/gtmb.2017.0278 29924656

[74] Looi K Megliorino R Shi FD Peng XX Chen Y Zhang JY. Humoral immune response to P16, a cyclin-dependent kinase inhibitor in human Malignancies. Oncol Rep. (2006) 16:1105–10. doi: 10.3892/or.16.5.1105 17016600

[75] Chen C Huang Y Zhang C Liu T Zheng HE Wan S. Circulating antibodies to P16 protein-derived peptides in breast cancer. Mol Clin Oncol. (2015) 3:591–4. doi: 10.3892/mco.2015.485 PMC4471576 26137272

[76] Dong X Yang M Sun H Lu J Zheng Z Li Z. Combined measurement of ca 15-3 with novel autoantibodies improves diagnostic accuracy for breast cancer. Onco Targets Ther. (2013) 6:273–9. doi: 10.2147/OTT.S43122 PMC3615893 23569391

[77] He X Jiang XH Yie KY Chen J Zhang JB Yie SM. An autoantibody against a 48-kd fragment of human DNA-topoiomerase I in breast cancer: implication for diagnosis and prognosis, and antibody-dependent cellular cytotoxicity in vitro. Cell Immunol. (2020) 347:104007. doi: 10.1016/j.cellimm.2019.104007 31732123

[78] Lacombe J Mange A Jarlier M Bascoul-Mollevi C Rouanet P Lamy PJ. Identification and validation of new autoantibodies for the diagnosis of dcis and node negative early-stage breast cancers. Int J Cancer. (2013) 132:1105–13. doi: 10.1002/ijc.27766 22886747

[79] Wang J Figueroa JD Wallstrom G Barker K Park JG Demirkan G. Plasma autoantibodies associated with basal-like breast cancers. Cancer Epidemiol Biomarkers Prev. (2015) 24:1332–40. doi: 10.1158/1055-9965.EPI-15-0047 PMC4560641 26070530

[80] Anderson KS Sibani S Wallstrom G Qiu J Mendoza EA Raphael J. Protein microarray signature of autoantibody biomarkers for the early detection of breast cancer. J Proteome Res. (2011) 10:85–96. doi: 10.1021/pr100686b 20977275 PMC3158028

[81] Henderson MC Silver M Tran Q Letsios EE Mulpuri R Reese DE. A noninvasive blood-based combinatorial proteomic biomarker assay to detect breast cancer in women over age 50 with bi-rads 3, 4, or 5 assessment. Clin Cancer Res. (2019) 25:142–9. doi: 10.1158/1078-0432.CCR-18-0843 30185421

[82] Lourenco AP Benson KL Henderson MC Silver M Letsios E Tran Q. A noninvasive blood-based combinatorial proteomic biomarker assay to detect breast cancer in women under the age of 50 years. Clin Breast Cancer. (2017) 17:516–25 e6. doi: 10.1016/j.clbc.2017.05.004 28624156

[83] Reese DE Henderson MC Silver M Mulpuri R Letsios E Tran Q. Breast density does not impact the ability of videssa(R) breast to detect breast cancer in women under age 50. PloS One. (2017) 12:e0186198. doi: 10.1371/journal.pone.0186198 29069101 PMC5656317

[84] Tabuchi Y Shimoda M Kagara N Naoi Y Tanei T Shimomura A. Protective effect of naturally occurring anti-her2 autoantibodies on breast cancer. Breast Cancer Res Treat. (2016) 157:55–63. doi: 10.1007/s10549-016-3801-4 27113738

[85] Demircan K Sun Q Bengtsson Y Seemann P Vallon-Christersson J Malmberg M. Autoimmunity to selenoprotein P predicts breast cancer recurrence. Redox Biol. (2022) 53:102346. doi: 10.1016/j.redox.2022.102346 35636018 PMC9157254

[86] Kulic A Sirotkovic-Skerlev M Jelisavac-Cosic S Herceg D Kovac Z Vrbanec D. Anti-P53 antibodies in serum: relationship to tumor biology and prognosis of breast cancer patients. Med Oncol. (2010) 27:887–93. doi: 10.1007/s12032-009-9301-1 19763913

[87] Ozmen T Gulluoglu BM Yegen CS Soran A. Autoimmune thyroid disease and breast cancer prognosis. J Breast Health. (2015) 11:67–71. doi: 10.5152/tjbh.2015.2462 28331694 PMC5351489

[88] Muller I Barrett-Lee PJ. The antigenic link between thyroid autoimmunity and breast cancer. Semin Cancer Biol. (2020) 64:122–34. doi: 10.1016/j.semcancer.2019.05.013 31128301

[89] Smyth PP. The thyroid, iodine and breast cancer. Breast Cancer Res. (2003) 5:235–8. doi: 10.1186/bcr638 PMC314438 12927031

[90] Bray F Ferlay J Soerjomataram I Siegel RL Torre LA Jemal A. Global cancer statistics 2018: globocan estimates of incidence and mortality worldwide for 36 cancers in 185 countries. CA Cancer J Clin. (2018) 68:394–424. doi: 10.3322/caac.21492 30207593

[91] Bach PB Mirkin JN Oliver TK Azzoli CG Berry DA Brawley OW. Benefits and harms of ct screening for lung cancer: A systematic review. JAMA. (2012) 307:2418–29. doi: 10.1001/jama.2012.5521 PMC3709596 22610500

[92] Liu Z Zhang F Jiang J Zhao C Zhu L Liu C. Early detection of lung cancer in a real-world cohort via tumor-associated immune autoantibody and imaging combination. Front Oncol. (2023) 13:1166894. doi: 10.3389/fonc.2023.1166894 37081975 PMC10110964

[93] Huang H Luo W Ni Y Sun S Wang C Zhang L. The diagnostic efficiency of seven autoantibodies in lung cancer. Eur J Cancer Prev. (2020) 29:315–20. doi: 10.1097/CEJ.0000000000000559 31764214

[94] Marmor HN Zorn JT Deppen SA Massion PP Grogan EL. Biomarkers in lung cancer screening: A narrative review. Curr Chall Thorac Surg. (2023) 5:5. doi: 10.21037/ccts-20-171 37016707 PMC10069480

[95] Auger C Moudgalya H Neely MR Stephan JT Tarhoni I Gerard D. Development of a novel circulating autoantibody biomarker panel for the identification of patients with ‘Actionable’ Pulmonary nodules. Cancers (Basel). (2023) 15(8):2259. doi: 10.3390/cancers15082259 37190187 PMC10136536

[96] Yang B Li X Ren T Yin Y. Autoantibodies as diagnostic biomarkers for lung cancer: A systematic review. Cell Death Discovery. (2019) 5:126. doi: 10.1038/s41420-019-0207-1 31396403 PMC6683200

[97] Qin J Zeng N Yang T Wan C Chen L Shen Y. Diagnostic value of autoantibodies in lung cancer: A systematic review and meta-analysis. Cell Physiol Biochem. (2018) 51:2631–46. doi: 10.1159/000495935 30562746

[98] Wu L Chang W Zhao J Yu Y Tan X Su T. Development of autoantibody signatures as novel diagnostic biomarkers of non-small cell lung cancer. Clin Cancer Res. (2010) 16:3760–8. doi: 10.1158/1078-0432.CCR-10-0193 20501620

[99] Doseeva V Colpitts T Gao G Woodcock J Knezevic V. Performance of a multiplexed dual analyte immunoassay for the early detection of non-small cell lung cancer. J Transl Med. (2015) 13:55. doi: 10.1186/s12967-015-0419-y 25880432 PMC4335536

[100] Ren S Zhang S Jiang T He Y Ma Z Cai H. Early detection of lung cancer by using an autoantibody panel in Chinese population. Oncoimmunology. (2018) 7:e1384108. doi: 10.1080/2162402X.2017.1384108 29308305 PMC5749655

[101] Boyle P Chapman CJ Holdenrieder S Murray A Robertson C Wood WC. Clinical validation of an autoantibody test for lung cancer. Ann Oncol. (2011) 22:383–9. doi: 10.1093/annonc/mdq361 PMC3030465 20675559

[102] Chapman CJ Healey GF Murray A Boyle P Robertson C Peek LJ. Earlycdt(R)-lung test: improved clinical utility through additional autoantibody assays. Tumour Biol. (2012) 33:1319–26. doi: 10.1007/s13277-012-0379-2 PMC3460172 22492236

[103] Jett JR Peek LJ Fredericks L Jewell W Pingleton WW Robertson JF. Audit of the autoantibody test, earlycdt(R)-lung, in 1600 patients: an evaluation of its performance in routine clinical practice. Lung Cancer. (2014) 83:51–5. doi: 10.1016/j.lungcan.2013.10.008 24268382

[104] Lam S Boyle P Healey GF Maddison P Peek L Murray A. Earlycdt-lung: an immunobiomarker test as an aid to early detection of lung cancer. Cancer Prev Res (Phila). (2011) 4:1126–34. doi: 10.1158/1940-6207.CAPR-10-0328 21733826

[105] Park Y Kim Y Lee JH Lee EY Kim HS. Usefulness of serum anti-P53 antibody assay for lung cancer diagnosis. Arch Pathol Lab Med. (2011) 135:1570–5. doi: 10.5858/arpa.2010-0717-OA 22129186

[106] Zhang Y Ying X Han S Wang J Zhou X Bai E. Autoantibodies against insulin-like growth factor−Binding protein-2 as a serological biomarker in the diagnosis of lung cancer. Int J Oncol. (2013) 42:93–100. doi: 10.3892/ijo.2012.1699 23165420 PMC3583617

[107] Wang J Shivakumar S Barker K Tang Y Wallstrom G Park JG. Comparative study of autoantibody responses between lung adenocarcinoma and benign pulmonary nodules. J Thorac Oncol. (2016) 11:334–45. doi: 10.1016/j.jtho.2015.11.011 26896032

[108] Murray A Chapman CJ Healey G Peek LJ Parsons G Baldwin D. Technical validation of an autoantibody test for lung cancer. Ann Oncol. (2010) 21:1687–93. doi: 10.1093/annonc/mdp606 PMC2911202 20124350

[109] Qiu J Choi G Li L Wang H Pitteri SJ Pereira-Faca SR. Occurrence of autoantibodies to annexin I, 14-3-3 theta and lamr1 in prediagnostic lung cancer sera. J Clin Oncol. (2008) 26:5060–6. doi: 10.1200/JCO.2008.16.2388 PMC2652098 18794547

[110] Li N Holden VK Deepak J Todd NW Jiang F. Autoantibodies against tumor-associated antigens in sputum as biomarkers for lung cancer. Transl Oncol. (2021) 14:100991. doi: 10.1016/j.tranon.2020.100991 33333369 PMC7736713

[111] Huang H Yang Y Zhu Y Chen H Yang Y Zhang L. Blood protein biomarkers in lung cancer. Cancer Lett. (2022) 551:215886. doi: 10.1016/j.canlet.2022.215886 35995139

[112] Chen SS Li K Wu J Peng ZY Wang ZD Wang JC. Stem signatures associated antibodies yield early diagnosis and precise prognosis predication of patients with non-small cell lung cancer. J Cancer Res Clin Oncol. (2021) 147:223–33. doi: 10.1007/s00432-020-03325-4 PMC11802159 32691153

[113] Patel AJ Tan TM Richter AG Naidu B Blackburn JM Middleton GW. A highly predictive autoantibody-based biomarker panel for prognosis in early-stage nsclc with potential therapeutic implications. Br J Cancer. (2022) 126:238–46. doi: 10.1038/s41416-021-01572-x PMC8770460 34728792

[114] Dekker E Tanis PJ Vleugels JLA Kasi PM Wallace MB. Colorectal cancer. Lancet. (2019) 394:1467–80. doi: 10.1016/S0140-6736(19)32319-0 31631858

[115] Kuipers EJ Grady WM Lieberman D Seufferlein T Sung JJ Boelens PG. Colorectal cancer. Nat Rev Dis Primers. (2015) 1:15065. doi: 10.1038/nrdp.2015.65 27189416 PMC4874655

[116] Brenner H Kloor M Pox CP. Colorectal cancer. Lancet. (2014) 383:1490–502. doi: 10.1016/S0140-6736(13)61649-9 24225001

[117] Shaukat A Levin TR. Current and future colorectal cancer screening strategies. Nat Rev Gastroenterol Hepatol. (2022) 19:521–31. doi: 10.1038/s41575-022-00612-y PMC9063618 35505243

[118] Simon K. Colorectal cancer development and advances in screening. Clin Interv Aging. (2016) 11:967–76. doi: 10.2147/CIA.S109285 PMC4958365 27486317

[119] Andre N Schmiegel W. Chemoradiotherapy for colorectal cancer. Gut. (2005) 54:1194–202. doi: 10.1136/gut.2004.062745 PMC1774863 16009693

[120] Ren G Li R Zheng G Du K Dan H Wu H. Prognostic value of normal levels of preoperative tumor markers in colorectal cancer. Sci Rep. (2023) 13:22830. doi: 10.1038/s41598-023-49832-5 38129505 PMC10739851

[121] Gao Y Wang J Zhou Y Sheng S Qian SY Huo X. Evaluation of serum cea, ca19-9, ca72-4, ca125 and ferritin as diagnostic markers and factors of clinical parameters for colorectal cancer. Sci Rep. (2018) 8:2732. doi: 10.1038/s41598-018-21048-y 29426902 PMC5807317

[122] Luo H Shen K Li B Li R Wang Z Xie Z. Clinical significance and diagnostic value of serum nse, cea, ca19-9, ca125 and ca242 levels in colorectal cancer. Oncol Lett. (2020) 20:742–50. doi: 10.3892/ol.2020.11633 PMC7286116 32566000

[123] Tong G Xu W Zhang G Liu J Zheng Z Chen Y. The role of tissue and serum carcinoembryonic antigen in stages I to iii of colorectal cancer-a retrospective cohort study. Cancer Med. (2018) 7:5327–38. doi: 10.1002/cam4.1814 PMC6246925 30302946

[124] Campos-da-Paz M Dorea JG Galdino AS Lacava ZGM de Fatima Menezes Almeida Santos M. Carcinoembryonic antigen (Cea) and hepatic metastasis in colorectal cancer: update on biomarker for clinical and biotechnological approaches. Recent Pat Biotechnol. (2018) 12:269–79. doi: 10.2174/1872208312666180731104244 30062978

[125] Rodriguez-Cobos J Vinal D Poves C Fernandez-Acenero MJ Peinado H Pastor-Morate D. Deltanp73, tap73 and delta133p53 extracellular vesicle cargo as early diagnosis markers in colorectal cancer. Cancers (Basel). (2021) 13(9):2240. doi: 10.3390/cancers13092240 34066954 PMC8124369

[126] Garranzo-Asensio M Guzman-Aranguez A Poves C Fernandez-Acenero MJ Montero-Calle A Ceron MA. The specific seroreactivity to ΔNp73 isoforms shows higher diagnostic ability in colorectal cancer patients than the canonical P73 protein. Sci Rep. (2019) 9:13547. doi: 10.1038/s41598-019-49960-x 31537884 PMC6753153

[127] Wu XJ Fang YJ Lin JZ Lu ZH Li LR Chen G. Circulating antibodies to carcinoembryonic antigen related to improved recurrence-free survival of patients with colorectal carcinoma. J Int Med Res. (2011) 39:838–45. doi: 10.1177/147323001103900317 21819716

[128] Babel I Barderas R Diaz-Uriarte R Martinez-Torrecuadrada JL Sanchez-Carbayo M Casal JI. Identification of tumor-associated autoantigens for the diagnosis of colorectal cancer in serum using high density protein microarrays. Mol Cell Proteomics. (2009) 8:2382–95. doi: 10.1074/mcp.M800596-MCP200 PMC2758763 19638618

[129] Konstadoulakis MM Syrigos KN Albanopoulos C Mayers G Golematis B. The presence of anti-carcinoembryonic antigen (Cea) antibodies in the sera of patients with gastrointestinal Malignancies. J Clin Immunol. (1994) 14:310–3. doi: 10.1007/BF01540984 7814460

[130] Montero-Calle A Garranzo-Asensio M Torrente-Rodriguez RM Ruiz-Valdepenas Montiel V Poves C Dziakova J. P53 and P63 proteoforms derived from alternative splicing possess differential seroreactivity in colorectal cancer with distinct diagnostic ability from the canonical proteins. Cancers (Basel). (2023) 15(7):2102. doi: 10.3390/cancers15072102 37046764 PMC10092954

[131] Silk AW Schoen RE Potter DM Finn OJ. Humoral immune response to abnormal muc1 in subjects with colorectal adenoma and cancer. Mol Immunol. (2009) 47:52–6. doi: 10.1016/j.molimm.2008.12.025 PMC2789300 19217667

[132] Pedersen JW Blixt O Bennett EP Tarp MA Dar I Mandel U. Seromic profiling of colorectal cancer patients with novel glycopeptide microarray. Int J Cancer. (2011) 128:1860–71. doi: 10.1002/ijc.25778 21344374

[133] Chen Y Lin P Qiu S Peng XX Looi K Farquhar MG. Autoantibodies to ca2+ Binding protein calnuc is a potential marker in colon cancer detection. Int J Oncol. (2007) 30:1137–44. doi: 10.3892/ijo.30.5.1137 17390015

[134] Liu W Wang P Li Z Xu W Dai L Wang K. Evaluation of tumour-associated antigen (Taa) miniarray in immunodiagnosis of colon cancer. Scand J Immunol. (2009) 69:57–63. doi: 10.1111/j.1365-3083.2008.02195.x 19140877

[135] Zhang JY Casiano CA Peng XX Koziol JA Chan EK Tan EM. Enhancement of antibody detection in cancer using panel of recombinant tumor-associated antigens. Cancer Epidemiol Biomarkers Prev. (2003) 12:136–43. 12582023

[136] Wang YQ Zhang HH Liu CL Xia Q Wu H Yu XH. Correlation between auto-antibodies to survivin and muc1 variable number tandem repeats in colorectal cancer. Asian Pac J Cancer Prev. (2012) 13:5557–62. doi: 10.7314/apjcp.2012.13.11.5557 23317217

[137] Chen JS Chen KT Fan WC Yu JS Chang YS Chan EC. Combined analysis of survivin autoantibody and carcinoembryonic antigen biomarkers for improved detection of colorectal cancer. Clin Chem Lab Med. (2010) 48:719–25. doi: 10.1515/CCLM.2010.123 20178447

[138] Fan CW Kuo YB Lin GP Chen SM Chang SH Li BA. Development of a multiplexed tumor-associated autoantibody-based blood test for the detection of colorectal cancer. Clin Chim Acta. (2017) 475:157–63. doi: 10.1016/j.cca.2017.10.022 29074220

[139] Chen H Werner S Butt J Zornig I Knebel P Michel A. Prospective evaluation of 64 serum autoantibodies as biomarkers for early detection of colorectal cancer in a true screening setting. Oncotarget. (2016) 7:16420–32. doi: 10.18632/oncotarget.7500 PMC4941325 26909861

[140] Liu W Li Z Xu W Wang Q Yang S. Humoral autoimmune response to igf2 mrna-binding protein (Imp2/P62) and its tissue-specific expression in colon cancer. Scand J Immunol. (2013) 77:255–60. doi: 10.1111/sji.12032 23421499

[141] Zhang JY Chan EK Peng XX Lu M Wang X Mueller F. Autoimmune responses to mrna binding proteins P62 and koc in diverse Malignancies. Clin Immunol. (2001) 100:149–56. doi: 10.1006/clim.2001.5048 11465943

[142] Chen JS Kuo YB Chou YP Chan CC Fan CW Chen KT. Detection of autoantibodies against rabphilin-3a-like protein as a potential biomarker in patient’s sera of colorectal cancer. Clin Chim Acta. (2011) 412:1417–22. doi: 10.1016/j.cca.2011.04.020 21536019

[143] Negm OH Hamed MR Schoen RE Whelan RL Steele RJ Scholefield J. Human blood autoantibodies in the detection of colorectal cancer. PloS One. (2016) 11:e0156971. doi: 10.1371/journal.pone.0156971 27383396 PMC4934916

[144] Chan CC Fan CW Kuo YB Chen YH Chang PY Chen KT. Multiple serological biomarkers for colorectal cancer detection. Int J Cancer. (2010) 126:1683–90. doi: 10.1002/ijc.24912 19795454

[145] Scanlan MJ Welt S Gordon CM Chen YT Gure AO Stockert E. Cancer-related serological recognition of human colon cancer: identification of potential diagnostic and immunotherapeutic targets. Cancer Res. (2002) 62:4041–7. 12124339

[146] Li Y Song R Li X Xu F. Expression and immunogenicity of ny-eso-1 in colorectal cancer. Exp Ther Med. (2017) 13:3581–5. doi: 10.3892/etm.2017.4405 PMC5450808 28588683

[147] Fan CW Chan CC Chen KT Twu J Huang YS Han CL. Identification of sec61beta and its autoantibody as biomarkers for colorectal cancer. Clin Chim Acta. (2011) 412:887–93. doi: 10.1016/j.cca.2011.01.012 21255561

[148] He Y Mou Z Li W Liu B Fu T Zhao S. Identification of impdh2 as a tumor-associated antigen in colorectal cancer using immunoproteomics analysis. Int J Colorectal Dis. (2009) 24:1271–9. doi: 10.1007/s00384-009-0759-2 19597826

[149] Raiter A Vilkin A Gingold R Levi Z Halpern M Niv Y. The presence of anti-grp78 antibodies in the serum of patients with colorectal carcinoma: A potential biomarker for early cancer detection. Int J Biol Markers. (2014) 29:e431–5. doi: 10.5301/jbm.5000086 24803280

[150] Tsunemi S Nakanishi T Fujita Y Bouras G Miyamoto Y Miyamoto A. Proteomics-based identification of a tumor-associated antigen and its corresponding autoantibody in gastric cancer. Oncol Rep. (2010) 23:949–56. doi: 10.3892/or_00000719 20204278

[151] Niloofa R De Zoysa MI Seneviratne LS. Autoantibodies in the diagnosis, prognosis, and prediction of colorectal cancer. J Cancer Res Ther. (2021) 17:819–33. doi: 10.4103/jcrt.JCRT_64_19 34528528

[152] Wang H Li X Zhou D Huang J. Autoantibodies as biomarkers for colorectal cancer: A systematic review, meta-analysis, and bioinformatics analysis. Int J Biol Markers. (2019) 34:334–47. doi: 10.1177/1724600819880906 31588830

[153] Kumamoto K Ishida H Kuwabara K Amano K Chika N Okada N. Clinical significance of serum anti-P53 antibody expression following curative surgery for colorectal cancer. Mol Clin Oncol. (2017) 7:595–600. doi: 10.3892/mco.2017.1368 28855992 PMC5574157

[154] Teras LR Gapstur SM Maliniak ML Jacobs EJ Gansler T Michel A. Prediagnostic antibodies to serum P53 and subsequent colorectal cancer. Cancer Epidemiol Biomarkers Prev. (2018) 27:219–23. doi: 10.1158/1055-9965.EPI-17-0407 29254936

[155] Wu J Qiu T Pan P Yu D Ju Z Qu X. Detection of serum anti-P53 antibodies from patients with colorectal cancer in China using a combination of P53- and phage-elisa: correlation to clinical parameters. Asian Pac J Cancer Prev. (2011) 12:2921–4. 22393964

[156] Pedersen JW Gentry-Maharaj A Fourkala EO Dawnay A Burnell M Zaikin A. Early detection of cancer in the general population: A blinded case-control study of P53 autoantibodies in colorectal cancer. Br J Cancer. (2013) 108:107–14. doi: 10.1038/bjc.2012.517 PMC3553520 23169294

[157] Suppiah A Alabi A Madden L Hartley JE Monson JR Greenman J. Anti-P53 autoantibody in colorectal cancer: prognostic significance in long-term follow-up. Int J Colorectal Dis. (2008) 23:595–600. doi: 10.1007/s00384-008-0458-4 18330580

[158] Deyoung MP Ellisen LW. P63 and P73 in human cancer: defining the network. Oncogene. (2007) 26:5169–83. doi: 10.1038/sj.onc.1210337 17334395

[159] Koziol JA Zhang JY Casiano CA Peng XX Shi FD Feng AC. Recursive partitioning as an approach to selection of immune markers for tumor diagnosis. Clin Cancer Res. (2003) 9:5120–6. 14613989

[160] Alvarez-Fernandez SM Barbariga M Cannizzaro L Cannistraci CV Hurley L Zanardi A. Serological immune response against adam10 pro-domain is associated with favourable prognosis in stage iii colorectal cancer patients. Oncotarget. (2016) 7:80059–76. doi: 10.18632/oncotarget.11181 PMC5346771 27517630

[161] Zhao F Cao M Jiang XH Xie K Ye SR Yie SM. A specific autoantibody against a novel tumour-association antigen derived from human DNA-topoiomerase I is a potential biomarker for early diagnosis and favourable prognosis in patients with colorectal carcinoma. Biomarkers. (2020) 25:149–56. doi: 10.1080/1354750X.2020.1714734 31922440

[162] Tang R Ko MC Wang JY Changchien CR Chen HH Chen JS. Humoral response to P53 in human colorectal tumors: A prospective study of 1,209 patients. Int J Cancer. (2001) 94:859–63. doi: 10.1002/ijc.1541 11745489

[163] Abe S Kawai K Ishihara S Nozawa H Hata K Kiyomatsu T. Prognostic value of pre- and postoperative anti-P53 antibody levels in colorectal cancer patients: A retrospective study. Oncology. (2017) 92:31–8. doi: 10.1159/000449527 27794579

[164] Kanojia D Garg M Gupta S Gupta A Suri A. Sperm-associated antigen 9 is a novel biomarker for colorectal cancer and is involved in tumor growth and tumorigenicity. Am J Pathol. (2011) 178:1009–20. doi: 10.1016/j.ajpath.2010.11.047 PMC3069833 21356354

[165] Fleshner K Carlsson SV Roobol MJ. The effect of the uspstf psa screening recommendation on prostate cancer incidence patterns in the USA. Nat Rev Urol. (2017) 14:26–37. doi: 10.1038/nrurol.2016.251 27995937 PMC5341610

[166] Aladwani M Lophatananon A Ollier W Muir K. Prediction models for prostate cancer to be used in the primary care setting: A systematic review. BMJ Open. (2020) 10:e034661. doi: 10.1136/bmjopen-2019-034661 PMC7371149 32690501

[167] Jayakrishnan R Schafer C Tan SH. Prostate cancer autoantibodies - applications in diagnosis, prognosis, monitoring disease progression and immunotherapy. Am J Clin Exp Urol. (2023) 11:79–102. 37168942 PMC10165224

[168] Sreekumar A Laxman B Rhodes DR Bhagavathula S Harwood J Giacherio D. Humoral immune response to alpha-methylacyl-coa racemase and prostate cancer. J Natl Cancer Inst. (2004) 96:834–43. doi: 10.1093/jnci/djh145 15173267

[169] Bradley SV Oravecz-Wilson KI Bougeard G Mizukami I Li L Munaco AJ. Serum antibodies to huntingtin interacting protein-1: A new blood test for prostate cancer. Cancer Res. (2005) 65:4126–33. doi: 10.1158/0008-5472.CAN-04-4658 15899803

[170] Dai L Li J Ortega R Qian W Casiano CA Zhang JY. Preferential autoimmune response in prostate cancer to cyclin B1 in a panel of tumor-associated antigens. J Immunol Res. (2014) 2014:827827. doi: 10.1155/2014/827827 24860838 PMC4016862

[171] O’Rourke DJ DiJohnson DA Caiazzo RJ Jr. Nelson JC Ure D O’Leary MP. Autoantibody signatures as biomarkers to distinguish prostate cancer from benign prostatic hyperplasia in patients with increased serum prostate specific antigen. Clin Chim Acta. (2012) 413:561–7. doi: 10.1016/j.cca.2011.11.027 PMC3268872 22146597

[172] Potluri HK Ng TL Newton MA Zhang J Maher CA Nelson PS. Antibody profiling of patients with prostate cancer reveals differences in antibody signatures among disease stages. J Immunother Cancer. (2020) 8(2):e001510. doi: 10.1136/jitc-2020-001510 33335027 PMC7745697

[173] Ummanni R Duscharla D Barett C Venz S Schlomm T Heinzer H. Prostate cancer-associated autoantibodies in serum against tumor-associated antigens as potential new biomarkers. J Proteomics. (2015) 119:218–29. doi: 10.1016/j.jprot.2015.02.005 25724726

[174] Schipper M Wang G Giles N Ohrnberger J. Novel prostate cancer biomarkers derived from autoantibody signatures. Transl Oncol. (2015) 8:106–11. doi: 10.1016/j.tranon.2015.02.003 PMC4415116 25926076

[175] Mintz PJ Rietz AC Cardo-Vila M Ozawa MG Dondossola E Do KA. Discovery and horizontal follow-up of an autoantibody signature in human prostate cancer. Proc Natl Acad Sci U.S.A. (2015) 112:2515–20. doi: 10.1073/pnas.1500097112 PMC4345585 25675522

[176] Xie C Kim HJ Haw JG Kalbasi A Gardner BK Li G. A novel multiplex assay combining autoantibodies plus psa has potential implications for classification of prostate cancer from non-malignant cases. J Transl Med. (2011) 9:43. doi: 10.1186/1479-5876-9-43 21504557 PMC3102624

[177] Zaenker P Ziman MR. Serologic autoantibodies as diagnostic cancer biomarkers–a review. Cancer Epidemiol Biomarkers Prev. (2013) 22:2161–81. doi: 10.1158/1055-9965.EPI-13-0621 24057574

[178] Liu W Peng B Lu Y Xu W Qian W Zhang JY. Autoantibodies to tumor-associated antigens as biomarkers in cancer immunodiagnosis. Autoimmun Rev. (2011) 10:331–5. doi: 10.1016/j.autrev.2010.12.002 PMC3119819 21167321

[179] Rastogi A Ali A Tan SH Banerjee S Chen Y Cullen J. Autoantibodies against oncogenic erg protein in prostate cancer: potential use in diagnosis and prognosis in a panel with C-myc, amacr and herv-K gag. Genes Cancer. (2016) 7:394–413. doi: 10.18632/genesandcancer.126 28191285 PMC5302040

[180] Rao DS Hyun TS Kumar PD Mizukami IF Rubin MA Lucas PC. Huntingtin-interacting protein 1 is overexpressed in prostate and colon cancer and is critical for cellular survival. J Clin Invest. (2002) 110:351–60. doi: 10.1172/JCI15529 PMC151092 12163454

[181] Alsoe L Stacy JE Fossa A Funderud S Brekke OH Gaudernack G. Identification of prostate cancer antigens by automated high-throughput filter immunoscreening. J Immunol Methods. (2008) 330:12–23. doi: 10.1016/j.jim.2007.10.011 18045611

[182] Chen WS Haynes WA Waitz R Kamath K Vega-Crespo A Shrestha R. Autoantibody landscape in patients with advanced prostate cancer. Clin Cancer Res. (2020) 26:6204–14. doi: 10.1158/1078-0432.CCR-20-1966 PMC7710628 32967941

[183] Reis BS Jungbluth AA Frosina D Holz M Ritter E Nakayama E. Prostate cancer progression correlates with increased humoral immune response to a human endogenous retrovirus gag protein. Clin Cancer Res. (2013) 19:6112–25. doi: 10.1158/1078-0432.CCR-12-3580 24081977

[184] Pandey JP Namboodiri AM Kistner-Griffin E. A genetic variant of fcgammariiia is strongly associatedwith humoral immunity to cyclin B1 in African American patients with prostate cancer. Immunogenetics. (2013) 65:91–6. doi: 10.1007/s00251-012-0660-y 23114687

[185] Xu L Lee JR Hao S Ling XB Brooks JD Wang SX. Improved detection of prostate cancer using a magneto-nanosensor assay for serum circulating autoantibodies. PloS One. (2019) 14:e0221051. doi: 10.1371/journal.pone.0221051 31404106 PMC6690541

[186] Massoner P Lueking A Goehler H Hopfner A Kowald A Kugler KG. Serum-autoantibodies for discovery of prostate cancer specific biomarkers. Prostate. (2012) 72:427–36. doi: 10.1002/pros.21444 22012634

[187] Mintz PJ Kim J Do KA Wang X Zinner RG Cristofanilli M. Fingerprinting the circulating repertoire of antibodies from cancer patients. Nat Biotechnol. (2003) 21:57–63. doi: 10.1038/nbt774 12496764

[188] Popkirov S Ayzenberg I Hahn S Bauer J Denno Y Rieckhoff N. Rho-associated protein kinase 2 (Rock2): A new target of autoimmunity in paraneoplastic encephalitis. Acta Neuropathol Commun. (2017) 5:40. doi: 10.1186/s40478-017-0447-3 28554330 PMC5448146

[189] Nesterova MV Johnson N Cheadle C Bates SE Mani S Stratakis CA. Autoantibody cancer biomarker: extracellular protein kinase A. Cancer Res. (2006) 66:8971–4. doi: 10.1158/0008-5472.CAN-06-1049 16982736

[190] Awad MM Mastini C Blasco RB Mologni L Voena C Mussolin L. Epitope mapping of spontaneous autoantibodies to anaplastic lymphoma kinase (Alk) in non-small cell lung cancer. Oncotarget. (2017) 8:92265–74. doi: 10.18632/oncotarget.21182 PMC5696179 29190913

[191] Mondello P Mian M Pitini V Cuzzocrea S Sindoni A Galletti M. Thyroid hormone autoantibodies: are they a better marker to detect early thyroid damage in patients with hematologic cancers receiving tyrosine kinase inhibitor or immunoregulatory drug treatments? Curr Oncol. (2016) 23:e165–70. doi: 10.3747/co.23.3026 PMC4900836 27330353

[192] Pelaez-Garcia A Barderas R Torres S Hernandez-Varas P Teixido J Bonilla F. Fgfr4 role in epithelial-mesenchymal transition and its therapeutic value in colorectal cancer. PloS One. (2013) 8:e63695. doi: 10.1371/journal.pone.0063695 23696849 PMC3655941

[193] Chew NJ Lim Kam Sian TCC Nguyen EV Shin SY Yang J Hui MN. Evaluation of fgfr targeting in breast cancer through interrogation of patient-derived models. Breast Cancer Res. (2021) 23:82. doi: 10.1186/s13058-021-01461-4 34344433 PMC8336364

[194] Levine KM Ding K Chen L Oesterreich S. Fgfr4: A promising therapeutic target for breast cancer and other solid tumors. Pharmacol Ther. (2020) 214:107590. doi: 10.1016/j.pharmthera.2020.107590 32492514 PMC7494643

[195] Facchinetti F Hollebecque A Bahleda R Loriot Y Olaussen KA Massard C. Facts and new hopes on selective fgfr inhibitors in solid tumors. Clin Cancer Res. (2020) 26:764–74. doi: 10.1158/1078-0432.CCR-19-2035 PMC7024606 31585937

[196] Anderson KS Cramer DW Sibani S Wallstrom G Wong J Park J. Autoantibody signature for the serologic detection of ovarian cancer. J Proteome Res. (2015) 14:578–86. doi: 10.1021/pr500908n PMC4334299 25365139

[197] Liu D. Cancer biomarkers for targeted therapy. biomark Res. (2019) 7:25. doi: 10.1186/s40364-019-0178-7 31807308 PMC6857213

[198] Rathi A Kumar D Hasan GM Haque MM Hassan MI. Therapeutic targeting of pim kinase signaling in cancer therapy: structural and clinical prospects. Biochim Biophys Acta Gen Subj. (2021) 1865:129995. doi: 10.1016/j.bbagen.2021.129995 34455019

[199] Li W Xiao J Zhou X Xu M Hu C Xu X. Stk4 regulates tlr pathways and protects against chronic inflammation-related hepatocellular carcinoma. J Clin Invest. (2015) 125:4239–54. doi: 10.1172/JCI81203 PMC4639976 26457732

[200] Julien SG Dube N Hardy S Tremblay ML. Inside the human cancer tyrosine phosphatome. Nat Rev Cancer. (2011) 11:35–49. doi: 10.1038/nrc2980 21179176

[201] Bollu LR Mazumdar A Savage MI Brown PH. Molecular pathways: targeting protein tyrosine phosphatases in cancer. Clin Cancer Res. (2017) 23:2136–42. doi: 10.1158/1078-0432.CCR-16-0934 PMC5413367 28087641

[202] Garranzo-Asensio M Solis-Fernandez G Montero-Calle A Garcia-Martinez JM Fiuza MC Pallares P. Seroreactivity against tyrosine phosphatase ptprn links type 2 diabetes and colorectal cancer and identifies a potential diagnostic and therapeutic target. Diabetes. (2022) 71:497–510. doi: 10.2337/db20-1206 35040477

[203] Schuler PJ Harasymczuk M Visus C Deleo A Trivedi S Lei Y. Phase I dendritic cell P53 peptide vaccine for head and neck cancer. Clin Cancer Res. (2014) 20:2433–44. doi: 10.1158/1078-0432.CCR-13-2617 PMC4017234 24583792

[204] Zhang L Zhou X Sha H Xie L Liu B. Recent progress on therapeutic vaccines for breast cancer. Front Oncol. (2022) 12:905832. doi: 10.3389/fonc.2022.905832 35734599 PMC9207208

[205] Onodi F Maherzi-Mechalikh C Mougel A Ben Hamouda N Taboas C Gueugnon F. High therapeutic efficacy of a new survivin lsp-cancer vaccine containing cd4(+) and cd8(+) T-cell epitopes. Front Oncol. (2018) 8:517. doi: 10.3389/fonc.2018.00517 30483475 PMC6243131

[206] Gao T Cen Q Lei H. A review on development of muc1-based cancer vaccine. BioMed Pharmacother. (2020) 132:110888. doi: 10.1016/j.biopha.2020.110888 33113416

[207] Lin X Tang S Guo Y Tang R Li Z Pan X. Personalized neoantigen vaccine enhances the therapeutic efficacy of bevacizumab and anti-pd-1 antibody in advanced non-small cell lung cancer. Cancer Immunol Immunother. (2024) 73:26. doi: 10.1007/s00262-023-03598-x 38280084 PMC10821847

[208] Cen L Zhang Z Sun Y Wu N Shao J Qian Z. Efficacy of mage-A4 long peptide as a universal immunoprevention cancer vaccine. Cancer Cell Int. (2024) 24:232. doi: 10.1186/s12935-024-03421-2 38961429 PMC11223347

[209] Wang J Zhou K Zhu H Wei F Ma S Kan Y. Current status and progress of the development of prostate cancer vaccines. J Cancer. (2023) 14:835–42. doi: 10.7150/jca.80803 PMC10088880 37056394

[210] Wagner S Mullins CS Linnebacher M. Colorectal cancer vaccines: tumor-associated antigens vs neoantigens. World J Gastroenterol. (2018) 24:5418–32. doi: 10.3748/wjg.v24.i48.5418 PMC6319136 30622371

[211] Shahnazari M Samadi P Pourjafar M Jalali A. Therapeutic vaccines for colorectal cancer: the progress and future prospect. Int Immunopharmacol. (2020) 88:106944. doi: 10.1016/j.intimp.2020.106944 33182032

[212] Pilla L Ferrone S Maccalli C. Methods for improving the immunogenicity and efficacy of cancer vaccines. Expert Opin Biol Ther. (2018) 18:765–84. doi: 10.1080/14712598.2018.1485649 PMC8670419 29874943

[213] Valverde A Montero-Calle M Arévalo B San Segundo-Acosta P Serafín V Alonso-Navarro M. Phage-derived and aberrant halotag peptides immobilized on magnetic microbeads for amperometric biosensing of serum autoantibodies and Alzheimer’s disease diagnosis. Anal Sens. (2021) 1:161–5. doi: 10.1002/anse.202100024

[214] Ahmed MU Saaem I Wu PC Brown AS. Personalized diagnostics and biosensors: A review of the biology and technology needed for personalized medicine. Crit Rev Biotechnol. (2014) 34:180–96. doi: 10.3109/07388551.2013.778228 23607309

[215] Edelsberg J Weycker D Atwood M Hamilton-Fairley G Jett JR. Cost-effectiveness of an autoantibody test (Earlycdt-lung) as an aid to early diagnosis of lung cancer in patients with incidentally detected pulmonary nodules. PloS One. (2018) 13:e0197826. doi: 10.1371/journal.pone.0197826 29787590 PMC5963796

